# A colorimetric method to measure in vitro nitrogenase functionality for engineering nitrogen fixation

**DOI:** 10.1038/s41598-022-14453-x

**Published:** 2022-06-20

**Authors:** Lucía Payá-Tormo, Diana Coroian, Silvia Martín-Muñoz, Artavazd Badalyan, Robert T. Green, Marcel Veldhuizen, Xi Jiang, Gema López-Torrejón, Janneke Balk, Lance C. Seefeldt, Stefan Burén, Luis M. Rubio

**Affiliations:** 1 Centro de Biotecnología y Genómica de Plantas, Universidad Politécnica de Madrid, Instituto Nacional de Investigación y Tecnología Agraria y Alimentaria (INIA-CSIC), Campus de Montegancedo UPM, Crta M-40 km 38 Pozuelo de Alarcón, 28223 Madrid, Spain; 2 Departamento de Biotecnología-Biología Vegetal, Escuela Técnica Superior de Ingeniería Agronómica, Alimentaria y de Biosistemas, Universidad Politécnica de Madrid, 28040 Madrid, Spain; 3 Department of Chemistry and Biochemistry, Utah State University, Logan, UT USA; 4 Department of Biochemistry and Metabolism, John Innes Centre, Norwich, NR4 7UH UK; 5 School of Biological Sciences, University of East Anglia, Norwich, NR4 7TJ UK

**Keywords:** Metabolic engineering, Plant biotechnology

## Abstract

Biological nitrogen fixation (BNF) is the reduction of N_2_ into NH_3_ in a group of prokaryotes by an extremely O_2_-sensitive protein complex called nitrogenase. Transfer of the BNF pathway directly into plants, rather than by association with microorganisms, could generate crops that are less dependent on synthetic nitrogen fertilizers and increase agricultural productivity and sustainability. In the laboratory, nitrogenase activity is commonly determined by measuring ethylene produced from the nitrogenase-dependent reduction of acetylene (ARA) using a gas chromatograph. The ARA is not well suited for analysis of large sample sets nor easily adapted to automated robotic determination of nitrogenase activities. Here, we show that a reduced sulfonated viologen derivative (S_2_V^red^) assay can replace the ARA for simultaneous analysis of isolated nitrogenase proteins using a microplate reader. We used the S_2_V^red^ to screen a library of NifH nitrogenase components targeted to mitochondria in yeast. Two NifH proteins presented properties of great interest for engineering of nitrogen fixation in plants, namely NifM independency, to reduce the number of genes to be transferred to the eukaryotic host; and O_2_ resistance, to expand the half-life of NifH iron-sulfur cluster in a eukaryotic cell. This study established that NifH from *Dehalococcoides ethenogenes* did not require NifM for solubility, [Fe-S] cluster occupancy or functionality, and that NifH from *Geobacter sulfurreducens* was more resistant to O_2_ exposure than the other NifH proteins tested. It demonstrates that nitrogenase components with specific biochemical properties such as a wider range of O_2_ tolerance exist in Nature, and that their identification should be an area of focus for the engineering of nitrogen-fixing crops.

## Introduction

Although almost 80% of the atmosphere is composed of nitrogen gas (N_2_), crop productivity in modern agriculture is limited by biologically available nitrogen such as oxidized (*e.g.* NO_3_^−^) or reduced (*e.g.* NH_4_^+^) species^1^. Crop yield is increased using synthetic N-based fertilizers that are costly both economically and ecologically, due to the consumption of non-renewable energy resources, production of greenhouse gasses, and water and air pollution^2^. On the other hand, biological nitrogen fixation (BNF) is performed by selected prokaryotes (bacteria and archaea), named diazotrophs for their capacity to grow using N_2_ as the sole N source^3^. A diverse range of diazotrophs are found in Nature and can be classified according to their lifestyle as free living, symbiotic (mainly bacteria living within root nodules of legume plants, including pulse crops), and those that live in associative or endophytic relationship with other organisms. Three biotechnological approaches are currently being explored to reduce the application of N-based fertilizers to cereal crops by enhancing their access to BNF^4,5^. In the first strategy, bacteria naturally associated with cereals are engineered to improve their colonization ability, N_2_-fixing capabilities or NH_3_ release. In the other two strategies, the plants are instead genetically engineered to either generate new symbiotic relationships between the non-legume plant and N_2_-fixing bacteria, thus mimicking the legume-rhizobium natural symbiosis, or by direct transfer of the prokaryotic N_2_ fixation genes into the plant, to create a crop capable of fixing N_2_ without the requirement for symbiotic associations. Both approaches are ambitious and challenging. The new symbiotic relationship requires molecular signaling between the bacteria and plants to avoid an immune response, the formation of a nodule-like structure with a low-O_2_ environment and the productive exchange of nutrients between the plant and the bacteria. On the other hand, the transfer of the N_2_ fixation capability is complicated by the estimated number of required genes (ca. 10–20), the sensitivity of their products towards O_2_, the need to perform time consuming functional validations, and difficulty to troubleshoot pathway engineering in plants^6^.

Diazotrophs harbor a protein complex called nitrogenase that converts nitrogen (N_2_) into ammonia (NH_3_) in an intricate process requiring a large amount of energy in the form of ATP and low potential electrons^7^. Nitrogenase has two protein components: an α_2_β_2_ heterotetrameric dinitrogenase formed by the *ni*trogen *f*ixation (*nif*) *nifD and nifK* gene products, and a *nifH*-encoded homodimeric dinitrogenase reductase. During N_2_ to NH_3_ reduction, one NifH homodimer binds to each αβ half of the NifDK protein and, in an ATP-dependent reaction, transfers the electrons needed to break the N_2_ triple bond^8^. Nitrogenase requires a minimum of 16 ATP molecules and 8 electrons to convert one molecule of N_2_ into two molecules of NH_3_^9^. The electrons are funneled through three [Fe-S] clusters, starting at a [Fe_4_S_4_] cluster bridging the two subunits of NifH, via the P-cluster ([Fe_8_S_7_]) and finally to the iron molybdenum cofactor (FeMo-co, [MoFe_7_S_9_C-(*R*)-homocitrate]). The latter two clusters are located at each αβ half of the NifDK heterodimer^9^. All three metalloclusters are extremely O_2_-sensitive, which makes engineering nitrogenase in plants especially challenging.

**Figure 1:**
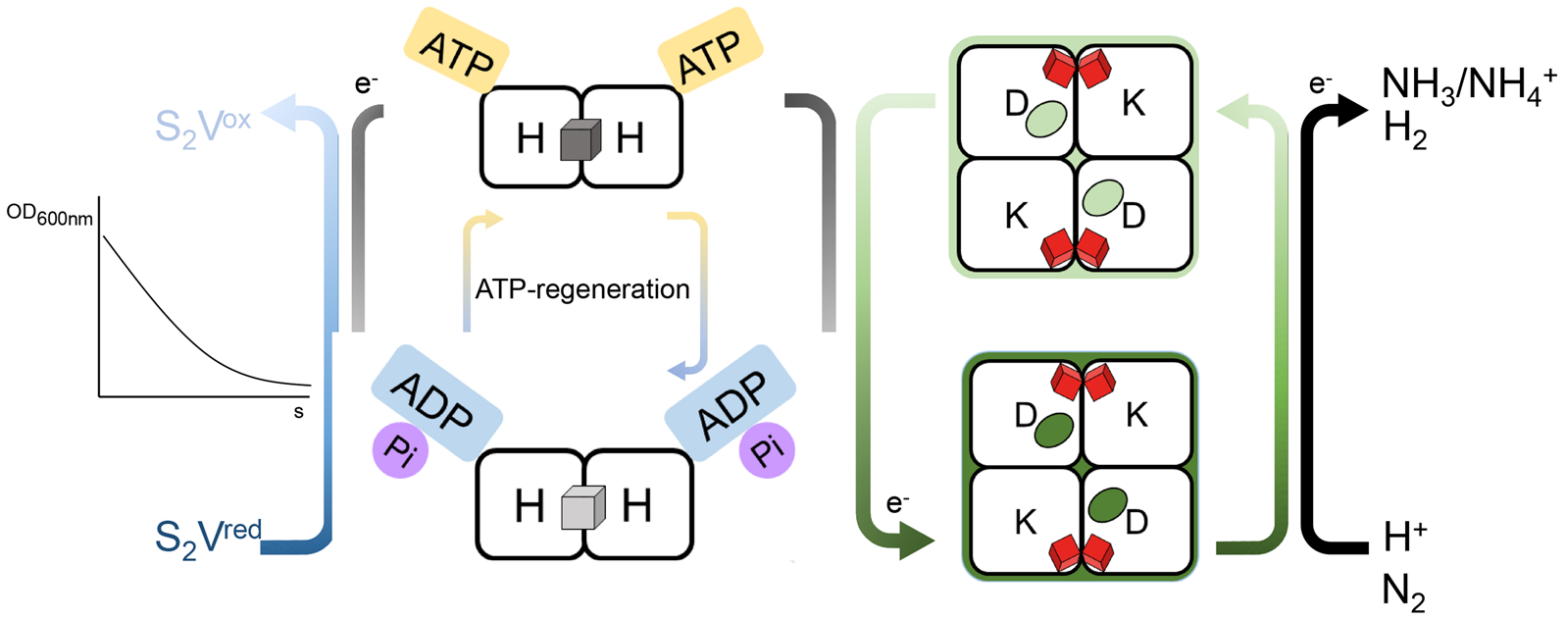
Schematic overview of nitrogenase activity and activity determination using S_2_V^red^. The flow of electrons from S_2_V^red^ to protons (H^+^) or N_2_ via NifH and NifDK is shown. At NifDK, H_2_ or NH_4_^+^ (the dominating form at pH 7) is produced. ATP is regenerated from ADP and phosphocreatine by the action of creatine phosphokinase. Nitrogenase activity is determined by measuring the decrease in absorbance at 600 nm.

The activity of nitrogenase can be determined in vitro (*e.g.* using pure protein components), in vivo (*e.g.* in free-living cells) or in situ (*e.g.* bacteria associated to plants) using various techniques. One direct method is the quantitative measurement of the ammonia produced using its natural substrate N_2_^10^_,_ or by ^15^N enrichment or ^15^N natural abundance methods^11–13^. Furthermore, nitrogenase activity can be indirectly measured as nitrogenase can also reduce protons into H_2_^14,15^, but also other double- and triple-bonded substrates such as acetylene, nitrite, nitrous oxide, and azide^16^. This promiscuity is often used by researchers to study nitrogenase in the laboratory. The most-used method is the acetylene reduction assay (ARA) in which acetylene is reduced to ethylene, which is detected using gas chromatography^17–20^. However, the ARA has several drawbacks. Firstly, the number of samples that can be measured is low due to the manual work involved. The manual steps include exchange of the gas phase in the reaction vial using inert argon to prevent N_2_ reduction; injection of acetylene to start the reaction; incubation in a water-bath, injection of EDTA or NaOH to stop the reaction; and finally injection of gas from the vial headspace into the gas chromatograph. Secondly, acetylene reduction cannot be easily monitored in real time, and only the end concentration of ethylene after a defined time is determined. This second limitation of the ARA was recently overcome by the development of a viologen-based electron donor to nitrogenase^21^. In that method a reduced sulfonated viologen derivative (1,1′-bis(3-sulfonatopropyl)-4,4′-bipyridinium radical, hereafter referred to as S_2_V^red^) replaces the function of sodium dithionite (DTH) as electron donor to NifH in vitro. Upon nitrogenase activity and turnover, this deeply violet-colored substrate is converted into an oxidized colorless form (S_2_V^ox^) with greatly diminished absorbance at 600 nm (Fig. 1). The decrease in absorbance over time is therefore linear with nitrogenase activity. However, the exposure of the violet-colored S_2_V^red^ to an oxidizing agent such as O_2_ (or other reactive oxygen species), or to other natural electron acceptors, will lead to its conversion into the oxidized and colorless form (S_2_V^ox^), limiting the use of S_2_V^red^ to in vitro measurements under anaerobic conditions.

In this work, we have adapted the S_2_V^red^ method to determine nitrogenase activity in 96-well microtiter plates with the aim to screen distinct NifH variants expressed in the mitochondria of the yeast *Saccharomyces cerevisiae* for functionality. The development of screening methods allows us to find Nif components with improved properties desirable for its expression in plant organelles. We demonstrate that: (1) the results obtained using S_2_V^red^ are in accordance with those seen when using the standard ARA; (2) the method is compatible with NifH proteins originating from different prokaryotic origins; and (3) many samples and assay conditions can be tested in parallel. We used S_2_V^red^ to determine the activity of nine distinct NifH proteins. NifH variants that were compatible with the *Azotobacter vinelandii* NifDK were tested for NifM dependency and O_2_ sensitivity, two properties of importance to engineer nitrogenase in crop plants.

## Results

### Adaptation and use of S_2_V^red^ for screening and activity determination of NifH proteins using 96 well microtiter plates

**Figure 2:**
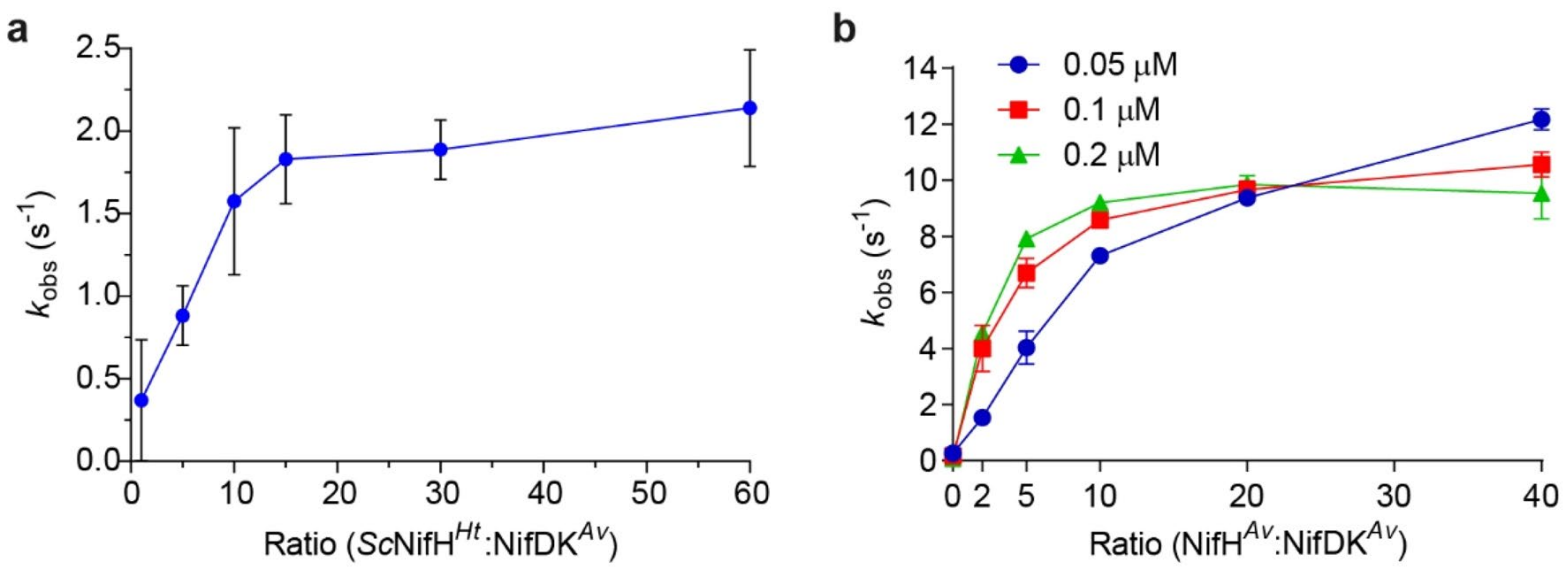
Adaptation and optimization of S_2_V^red^ for nitrogenase activity determination using 96-well microtiter plates. **(a)** Nitrogenase activity (*k*_obs_ (s^−1^)) measured in a cuvette using *Sc*NifH^*Ht*^ and NifDK^*Av*^ at 0–60 × molar ratios in reactions containing 0.4 µM NifDK^*Av*^. Mean and SD is shown. *n* = 2 technical replicates. **(b)** Nitrogenase activity (*k*_obs_ (s^−1^)) measured in a 96-well microtiter plate using NifH^*Av*^ and NifDK^*Av*^ at 0–40 × molar ratios in reactions containing 0.05 µM (blue dots), 0.1 µM (red squares) or 0.2 µM (green triangles) NifDK^*Av*^. Mean and SD is shown. *n* = 2 technical replicates.

To confirm that S_2_V^red^-dependent electron donation to nitrogenase is not unique to NifH isolated from *A. vinelandii* (hereafter denoted as NifH^*Av*^), as previously shown^21^, but is also suitable for functional screening of novel NifH variants, we combined the *Hydrogenobacter thermophilus* NifH protein previously isolated from *S. cerevisiae*^22^ with NifDK^*Av*^. NifH variants expressed in yeast are hereafter denoted as *Sc*NifH^*Xx*^ where *Sc* and *Xx* indicates *S. cerevisiae* and the species from which the NifH sequence was obtained, respectively. Robust, although slower, oxidation of S_2_V^red^ was observed using *Sc*NifH^*Ht*^ compared to NifH^*Av*^ (Fig. 2). This was expected from the lower specific activity of *Sc*NifH^*Ht*^ when combined with NifDK^*Av*^ determined by ARA in previous studies^22^. We next tested functionality of the S_2_V^red^ assay using NifH^*Av*^ and NifDK^*Av*^ in 96 well microtiter plates sealed in the glove box (95% N_2_ and 5% H_2_). Several NifDK concentrations (0.05–0.2 µM) and NifH to NifDK molar ratios (2x-40x) were tested to identify conditions that provided a robust and linear rate constant (*k*_obs_) over an extended time-period (to facilitate sample preparations and plate handling), and that minimized the amount of NifH and NifDK proteins required for the assay. Supplementary Fig. S1 shows the decrease in absorbance over time under the tested experimental conditions. As expected, higher NifDK concentrations resulted in a faster S_2_V^red^ oxidation (Supplementary Fig. S1d). Supplementary Figure S2 shows the corresponding *k*_obs_ variation over time. The fast S_2_V^red^ oxidation observed at high NifDK concentration resulted in lower *k*_obs_ (Supplementary Fig. S2c), presumably because the concentration of S_2_V^red^ was already suboptimal by the time the plate was being scanned (after sealing in the glove box and transfer to the plate reader). The maximum activity observed under the conditions tested here (*k*_obs_ of ca. 12 s^−1^) was obtained when the mixture contained 2 µM NifH^*Av*^ and 0.05 µM NifDK^*Av*^ (corresponding to a NifH:NifDK ratio of 40) (Fig. 2b), and was almost identical to that reported when performed under argon atmosphere and using cuvettes^21^. Importantly, the decrease in absorbance was linear under these conditions for more than 10 min (Supplementary Fig. S1b) (R^2^ = 0.9997, t_1_ = 1 to t_2_ = 10 min), which provided sufficient time for microtiter plate preparation, transfer and reading. These experimental conditions were therefore implemented for subsequent use of S_2_V^red^.

### Solubility screening of NifH variants targeted to the mitochondria of yeast

To obtain proof-of-concept of S_2_V^red^ suitability to quickly screen the function of engineered Nif proteins, we used a library of 35 mitochondria-targeted NifH proteins in yeast (Supplementary Tables S1 and S2). The bulk of the NifH variants originated from a library that had previously been tested in *Nicotiana benthamiana*^22^*.* The NifH variants were expressed as N-terminally TwinStrep (TS)-tagged proteins with a Cox4 mitochondria targeting signal^23,24^. The purpose of the TS-tag was to enable equal detection of the distinct NifH variants and their subsequent isolation using Strep-tag affinity chromatography (STAC). The NifH proteins were co-expressed with NifM, NifU and NifS using galactose inducible promoters. NifM has been proposed to be necessary for proper folding of the NifH polypeptide^25^, whereas NifU and NifS provide NifH with its [Fe_4_S_4_] cluster^7^. All the accessory proteins originated from *A. vinelandii* and were equipped with a Su9 leader sequence^24,26^. The functionality of the Cox4 and the Su9 sequences for mitochondrial targeting of each respective protein in yeast has been shown previously^22,27,28^.

**Figure 3:**
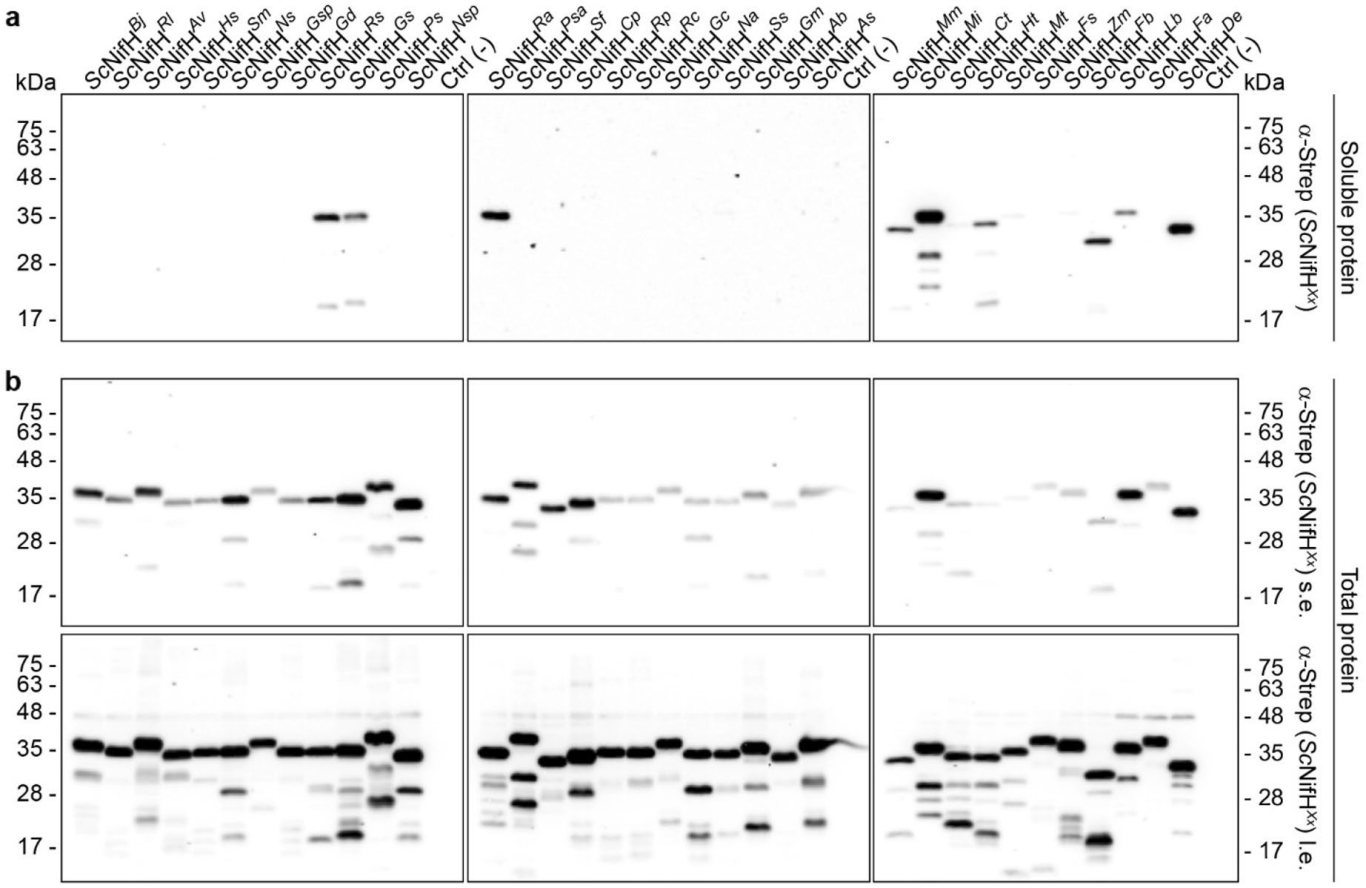
NifH library solubility screening. **(a,b)** Presence of *Sc*NifH^*Xx*^ (see Supplementary Table S2 for full species names) in soluble (a) and total (b) protein extracts was determined using antibodies detecting the TwinStrep-tag (α-Strep). Two different exposure times (s.e., short exposure (above) and l.e., long exposure (below)) are shown for the analysis of total extracts.

With the assumption that Nif components must accumulate as soluble proteins to be relevant for engineering nitrogenase in eukaryotes, we first tested the NifH variant solubility. Accumulation of *Sc*NifH^*Xx*^ variant polypeptides, co-expressed with NifM, NifU and NifS, was confirmed by immunoblot analysis of total protein extracts (Supplementary Fig. S3). Nine out of the 35 *Sc*NifH variants were detectable at noticeable levels in soluble extracts (Fig. 3). These nine NifH variants originated from *Roseiflexus* sp. (strain RS-1), *H. thermophilus* (strain TK-6), *Geobacter sulfurreducens* (strain PCA), *Ruminococcus albus* (strain SY3), *Methanothermobacter marburgensis* (strain Marburg), *Methanocaldococcus infernus* (strain ME), *Firmicutes* bacterium CAG:536, *Leptolyngbya boryana* (strain Dg5) and *Dehalococcoides ethenogenes* (strain 195). Of these nine, the NifH variants from *H. thermophilus*, *M. marburgensis* and *M. infernus* were shown to be soluble in a previous study from our group^22^.

### Isolation and activity measurements of soluble *Sc*NifH variants

**Figure 4:**
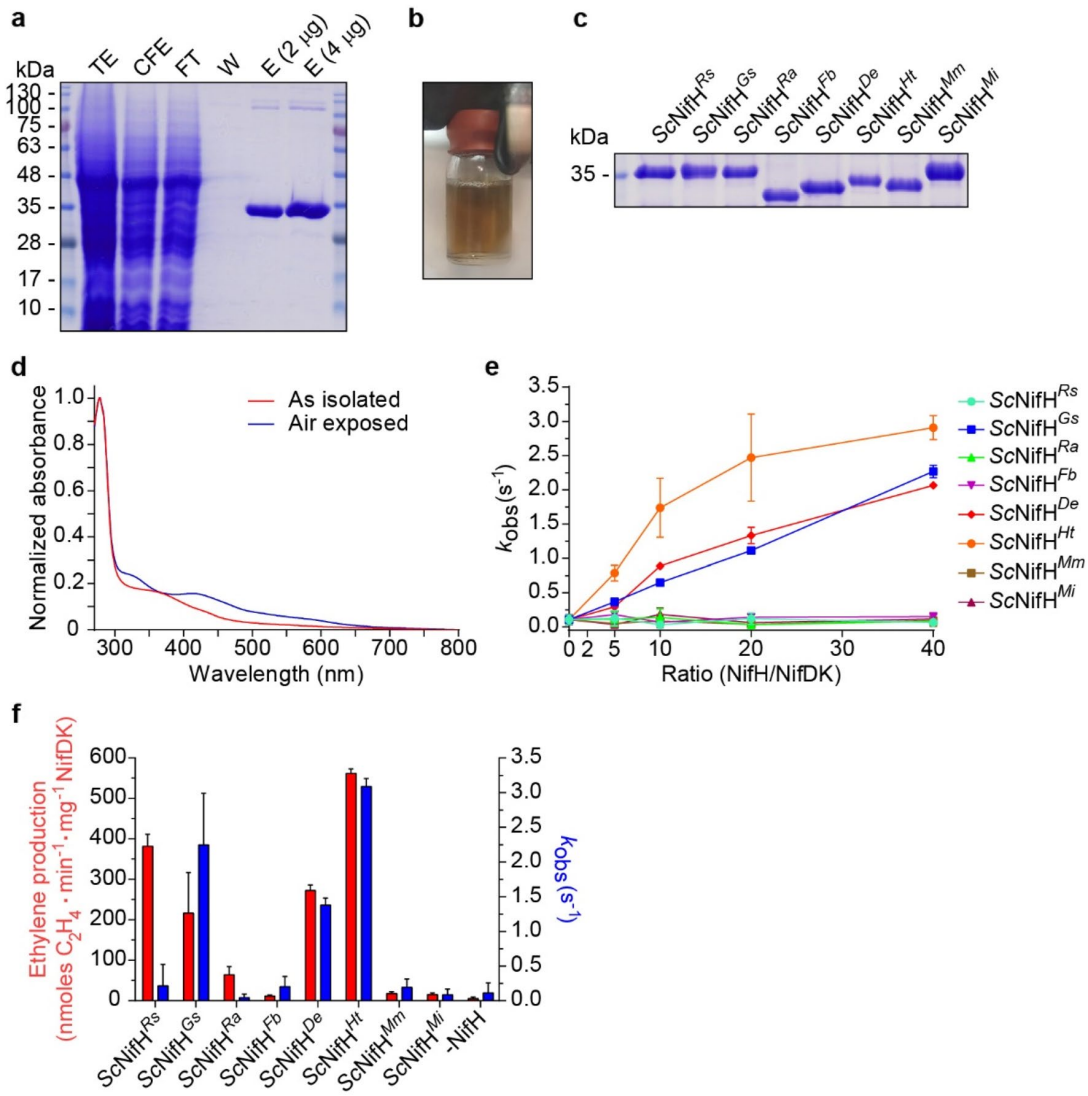
Functionality of soluble *Sc*NifH^*Xx*^ candidates. **(a)** Example of the STAC-purification process of *Sc*NifH^*Xx*^ (represented here by *Sc*NifH^*Ra*^). TE, total extract after yeast cell breakage using high-pressure homogenizer; CFE, cell-free extract after centrifugation and filtering of the TE; FT, flow-through after passing the CFE through the STAC column; W, wash fraction; E, final concentrated and desalted elution fraction. **(b)** Example of concentrated and desalted elution fraction (here represented by *Sc*NifH^*Fb*^, Supplementary Fig. S4d), 7 ml final volume. **(c)** Coomassie staining of soluble *Sc*NifH^*Xx*^ variants isolated from soluble yeast extracts using STAC. Approximately 3 µg protein was loaded per sample. More details of the purification process are shown in Supplementary Fig. S4. The uncropped Coomassie stained gel is shown in Fig. S11. **(d)** Example of UV–vis absorption spectra of as-isolated and air-exposed *Sc*NifH^*Xx*^ (represented here by *Sc*NifH^*Ra*^). **(e)** Nitrogenase activities with increasing concentrations of *Sc*NifH^*Xx*^ proteins using S_2_V^red^ as electron donor and NifDK^*Av*^ as electron acceptor. Mean and SD is shown. *n* = 2 technical replicates. **(f)** Nitrogenase activities using *Sc*NifH^*Xx*^ proteins and NifDK^*Av*^ (at a 40:1 molar ratio) as determined by ARA (ethylene production, left Y-axis, red bars) or using S_2_V^red^ (k_obs_^(s-1)^, right Y-axis, blue bars). Mean and SD is shown. *n* = 3 technical replicates (ARA) and *n* = 4 (S_2_V^red^).

Three of the nine soluble *Sc*NifH variants (from *H. thermophilus*, *M. marburgensis* and *M. infernus*) had already been purified in our laboratory^22^. The remaining *Sc*NifH proteins (together with *Sc*NifH^*Ht*^ that was reisolated for further work described in this study) were isolated using STAC under anaerobic conditions (Fig. 4a–c, Supplementary Fig. S4). The yield varied from 9–35 mg *Sc*NifH per 100 g cell paste for six of the variants (Table 1), in line to what was previously reported for *Sc*NifH^*Mm*^ and *Sc*NifH^*Mi*^. Only *Sc*NifH^*Lb*^ was isolated at much lower level. This variant was excluded from further analysis. These *Sc*NifH proteins all presented color and an UV–vis absorbance spectrum characteristic of [Fe–S] cluster containing proteins (Fig. 4d, Supplementary Fig. S5). Iron quantification suggested that six *Sc*NifH variants had a similar amount of bound [Fe-S] cluster as NifH^*Av*^ purified from its native host^29^ (Table 1), while *Sc*NifH^*Mi*^ and *Sc*NifH^*Mm*^ were isolated with much lower Fe content.

**Table 1:** Iron content per NifH dimer and purification yield for all soluble NifH variants purified from yeast.

NifH variant	Fe atoms per NifH dimer	Yield (mg NifH per 100 g cells)
*Roseiflexus* sp.	3.11 ± 0.42	35.2
*G. sulfurreducens*	2.52 ± 0.40	11.8
*R. albus*	3.22 ± 1.24	23.0
*D. ethenogenes*	1.93 ± 0.004	8.5
*D. ethenogenes (-NifM)*	1.97 ± 0.44	14.6
*Firmicutes bacterium*	2.79 ± 0.20	23.0
*H. thermophilus* (reisolated in this work)	2.06 ± 0.04	20.2
*M. marburgensis* (purified in previous work)	0.74 ± 0.41	2.86
*M. infernus* (purified in previous work)	0.87 ± 0.09	19.9
*L. boryana*	–	–
*A. vinelandii* (purified from *A. vinelandii*)	3.19 ± 0.05	–

Iron content shows mean and standard deviation (n = 2 technical replicates).

We then performed nitrogenase assays with the different *Sc*NifH proteins using S_2_V^red^ as the electron donor to NifH, and NifDK^*Av*^ as its electron acceptor (Fig. 4e). Three variants, namely those originating from *H. thermophilus* (*Sc*NifH^*Ht*^), *G. sulfurreducens* (*Sc*NifH^*Gs*^) and *D. ethenogenes* (*Sc*NifH^*De*^), accelerated S_2_V^red^ oxidation when the *Sc*NifH:NifDK^*Av*^ ratio was increased, which is expected from a functional and NifDK^*Av*^-compatible NifH variant. These *Sc*NifH proteins showed acetylene reduction activities consistent with those obtained using S_2_V^red^ (Fig. 4e,f). Interestingly, *Sc*NifH^*Rs*^ (and to some extent *Sc*NifH^*Ra*^) could act as reductase for NifDK^*Av*^ during ARA (*i. e.* when using DTH as electron donor), but not in the S_2_V^red^ assay (Fig. 4f). This divergence could potentially originate from different reduction potential requirements among the NifH variants, as S_2_V^red^ has a potential of − 0.40 V vs Normal Hydrogen Electrode (NHE)^21^ and DTH has a potential of − 0.66 V vs NHE^30^. Whether this discrepancy indicates a different mechanism or requirement for NifH^*Rs*^ activity remains to be investigated in future studies.

### Inhibition of *Sc*NifH by O_2_

**Figure 5:**
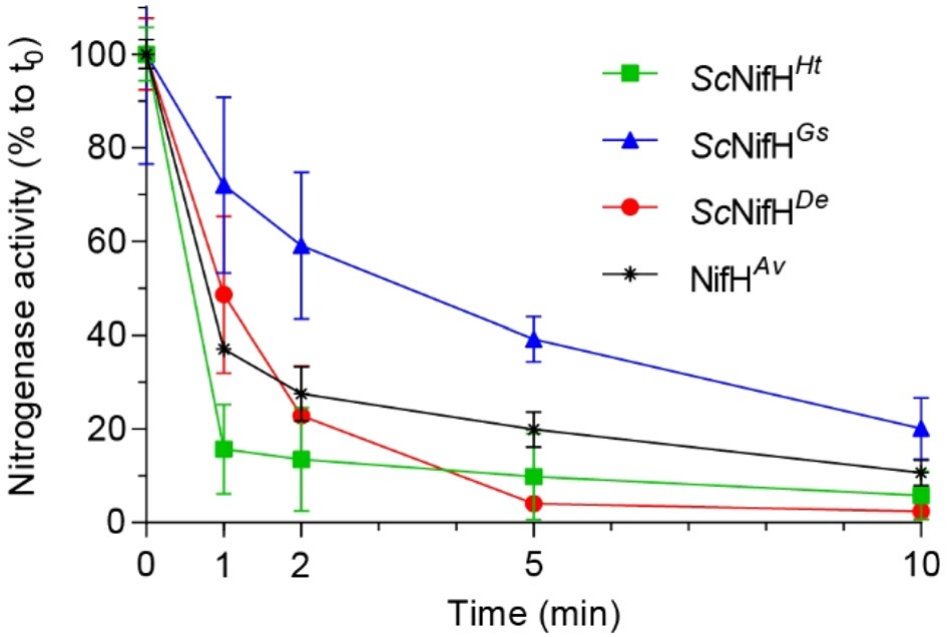
Sensitivity of *Sc*NifH^*Xx*^ variants to O_2_. Nitrogenase activity of *Sc*NifH^*Ht*^ (green squares), *Sc*NifH^*Gs*^ (blue triangles) and *Sc*NifH^*De*^ (red dots) was measured by ARA upon exposure to oxygen. NifH^*Av*^ was used as NifH control protein (black stars). The molar ratio of NifH:NifDK^*Av*^ was 40:1. Nitrogenase activity is shown in relation to the activity obtained prior to oxygen exposure at t_0_ (for *Sc*NifH^*Ht*^ 532 ± 30 units (nmol ethylene formed per min and mg of NifDK^*Av*^), for *Sc*NifH^*Gs*^ 159 ± 37 units, for *Sc*NifH^*De*^ 188 ± 14 units and for NifH^*Av*^ 1673 ± 51 units). Mean and SD is shown. *n* = 5 or 6 technical replicates.

The NifDK^*Av*^ compatible *Sc*NifH variants were assayed for their sensitivity to O_2_, which represents a major barrier to engineer nitrogenase in plants. As S_2_V^red^ itself is oxidized by O_2_, and O_2_-destruction of the [Fe_4_S_4_]-cluster at NifH is extremely fast (the half-life of NifH upon O_2_ exposure is reported to be about 30–45 s)^31,32^, we were not able to design an experiment using S_2_V^red^ to study the effect of O_2_ on the activity. We therefore measured the *Sc*NifH variants capacity to support acetylene reduction upon exposure to O_2_ following a previously reported method^31^. In short, DTH present in the buffer of the isolated *Sc*NifH protein was first removed using a desalting column inside an anaerobic glove box. The *Sc*NifH protein was then added to anaerobic buffer in a glass vial containing argon in the headspace (representing t = 0). Then, O_2_ was injected into the headspace to a final concentration of 20% and the vial was incubated with rigorous shaking. At distinct time points, *Sc*NifH was extracted using a Hamilton syringe and transferred to an open vial containing anaerobic buffer supplemented with DTH to quench the O_2_. Finally, NifDK^*Av*^ and an ATP-regenerating mixture was added before the standard ARA. Similar to the *Klebsiella pneumoniae* NifH protein^31^, the half-life for NifH^*Av*^ was less than one minute (Fig. 5). While *Sc*NifH^*Ht*^ and *Sc*NifH^*De*^ presented similar kinetics regarding the inhibition from O_2_ exposure as NifH^*Av*^, the *Sc*NifH^*Gs*^ retained 50% activity for about 4 min, and about 25% activity after 10 min O_2_ exposure.

### NifM-dependency for *Sc*NifH solubility and functionality

**Figure 6:**
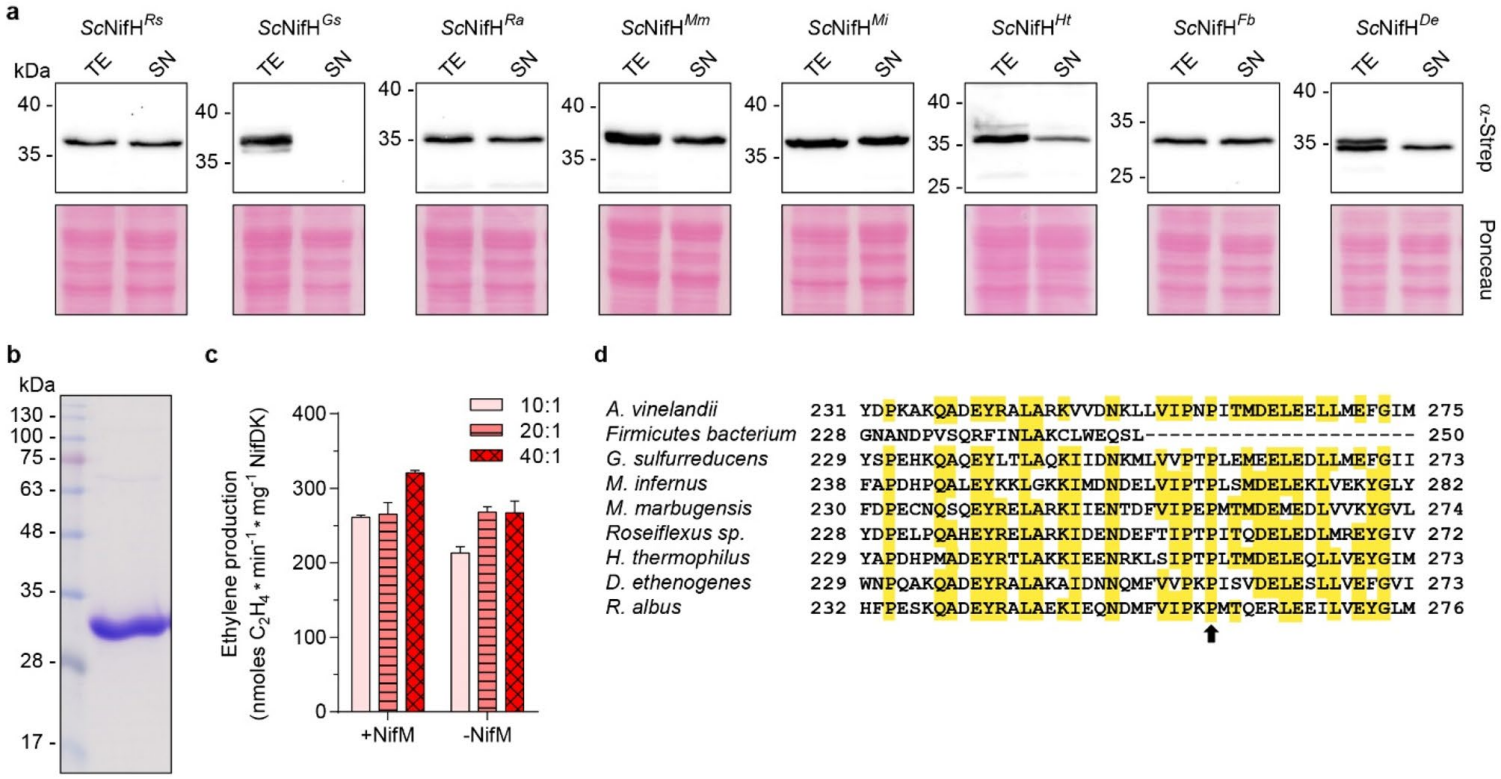
Effect of NifM^*Av*^ on *Sc*NifH^*Xx*^ solubility and functionality. **(a)** Immunoblot analysis of the levels of *Sc*NifH^*Xx*^ variants in total yeast extracts (TE) and the soluble fractions (SN) when expressed in the absence of NifM^*Av*^. The uncropped immunoblots and membranes are shown in Fig. S12. **(b)** *Sc*NifH^*De*^ isolated from yeast cells not expressing NifM^*Av*^. The uncropped Coomassie stained gel is shown in Fig. S13. **(c)** Comparison of the specific activity of *Sc*NifH^*De*^ isolated from yeast cells expressing (+ NifM) or not expressing NifM (− NifM) using ARA. The molar ratio of *Sc*NifH^*De*^ to NifDK^*Av*^ is indicated. Mean and SD is shown. *n* = 2 technical replicates. **(d)** Alignment of NifH^*Av*^ with the eight *Sc*NifH^*Xx*^ variants analyzed in (**a**). The C-terminal domain containing Pro^259^ in NifH^*Av*^ (indicated by a black arrow) proposed to be the target of NifM action is shown. The full sequence alignment can be found in Supplementary Fig. S7.

Co-expression of the *A. vinelandii nifM* gene with *nifH*^Av^ is required for the accumulation of functional NifH^*Av*^ protein in the mitochondria of *S. cerevisiae*^33^. Several of the NifH sequences in this study were selected because of the absence of a *nifM* orthologue in the organism’s genome. To test whether NifM was required for the solubility of the eight *Sc*NifH variants, we compared their accumulation in total and soluble protein extracts when co-expressed with NifU^*Av*^ and NifS^*Av*^, but not NifM^*Av*^, in yeast. Surprisingly, six of the eight *Sc*NifH variants (*Roseiflexus* sp., *R. albus*, *M. marburgensis*, *M. infernus*, *Firmicutes* bacterium, and *D. ethenogenes*) showed no obvious decrease in solubility when NifM^*Av*^ was absent (Fig. 6a). To test whether functionality could be affected although solubility was not, we isolated *Sc*NifH^*De*^ from the yeast strain not expressing NifM^*Av*^ (Fig. 6b, Supplementary Fig. S6a). The UV–vis spectrum suggested no apparent difference in [Fe-S] cluster content (Supplementary Fig. S6b), and the specific activity was similar to *Sc*NifH^*De*^ protein isolated from cells co-expressing NifM^*Av*^ (Fig. 6c).

Seven of our eight *Sc*NifH variants contained a proline residue at the site corresponding to Pro^259^ (when including the methionine) in *A. vinelandii* (Fig. 6d, Supplementary Fig. S7), which is thought to be the target of NifM prolyl isomerase activity^25^. The NifH protein from *Firmicutes* bacterium is shorter and terminates before this proline. Interestingly, the only genome of the eight selected NifH variants that contained a gene with high similarity to NifM^*Av*^ was *G. sulfurreducens* (Supplementary Table S2). *Sc*NifH^*Gs*^ was also the variant that was least soluble when NifM^*Av*^ was not co-expressed (Fig. 6a). The only other protein that showed reduced solubility in the absence of NifM^*Av*^ was *Sc*NifH^*Ht*^. The genome of *H. thermophilus* harbors a gene encoding a hypothetical protein with a PPIC-type PPIASE domain and with moderate similarity to NifM^*Av*^. Interestingly, isolation of the soluble population of *Sc*NifH^*Ht*^ that was produced in the absence of NifM^*Av*^ resulted in a protein with identical specific activity to *Sc*NifH^*Ht*^ isolated from yeast cells co-expressing of NifM^*Av*^ (Supplementary Fig. S8). Therefore, the direct action of NifM with regards to NifH is not clear and to some extent in disagreement with the published literature^7^, and should be the topic of future studies.

## Discussion

The transfer of prokaryotic nitrogenase activity into cereals could generate crops suited to grow well under limited nitrogen fertilizer. Although there are excellent reports on engineering of nitrogenase in heterologous (non-N_2_-fixing) bacterial hosts^34–40^, our experience is that it is very difficult to directly translate and transfer that knowledge to a eukaryotic system and expect comparable results, even in a relatively simple, unicellular eukaryote such as yeast^41^. A major challenge arises from the extremely complex biochemical requirements of the nitrogenase enzyme and its stepwise maturation involving several inter-dependent gene products^7^. Additionally, from a metabolic point of view, nitrogenase requires high levels of energy and reducing power in an environment that is low in O_2_ to protect its metalloclusters from oxidative damage.

In this study we have developed an important part of the nitrogenase engineering process, namely the analysis of NifH protein functionality in a high throughput assay. While the ARA is very precise, it requires training to generate consistent results, it is rather time-consuming and would be difficult to scale up for screening large numbers of samples and/or conditions. We optimized the S_2_V^red^ assay and showed that it fulfilled many of our main objectives, most importantly to be fast and simple to use, to require a lower amount of purified proteins (corresponding to about half of that used in the ARA as the reaction volume is smaller), and to not depend on expensive or sophisticated equipment. We also believe that this method is easily adaptable to automated robotic systems as the reactions are performed in microtiter plates. In addition, the S_2_V^red^ assay has two important advantages over the ARA. Firstly, activities can be monitored in real-time, which means that it is possible to directly study the effect of various effector molecules or reaction components on nitrogenase functionality. Secondly, the reduction potential of S_2_V^red^ (used as the electron donor to NifH) is much closer to that of ferredoxin or flavodoxin, the physiological reductants of nitrogenase^42,43^ than DTH. NifF for example, a flavodoxin in the diazotrophic free-living model-bacteria *A. vinelandii* donating electrons to NifH, harbors a flavin mononucleotide (FMN) cofactor with a redox potential in the semiquinone/hydroquinone state of − 0.483 V vs NHE^44^. The corresponding potentials for S_2_V^red^ is − 0.40 V vs NHE, compared to − 0.66 V vs NHE for DTH^21,30^. However, it is important to note that other reported flavodoxins and ferredoxins have lower reduction potentials, for example flavodoxin in *A. chroococcum* (− 522 mV)^45^ and ferredoxin in *A. vinelandii* (− 619 mV)^46^, and that S_2_V^red^ would not be a suitable electron donor to study nitrogenases requiring such strong reductants. Whether this could explain the lack of nitrogenase activity when combining *Sc*NifH^*Rs*^ and *Sc*NifH^*Ra*^ with NifDK^*Av*^ in the S_2_V^red^ is not clear, as it is also possible that other steric or charge factors prelude productive electron transfer from S_2_V^red^ to these NifH variants. Other important drawback with using S_2_V^red^ is that it is not commercially available, and that it cannot be used directly with yeast extracts as it is effectively oxidized by unknown molecule(s) in the lysate (data not shown). Therefore, Nif proteins must be purified prior to the activity assay. Solving this limitation would further expand the use of the S_2_V^red^.

Regarding the functional assessment of Nif proteins expressed in yeast and plants, we have observed that many of the essential Nif components have poor solubility, especially NifH and NifB^22,24,27,47^. This is critical as the structural components (NifH and NifDK) are needed at very high levels during nitrogen fixation. In N_2_-fixing *A. vinelandii* for example the NifH concentration within the cell can reach up to 100 μM^48^, whereas in *K. oxytoca* about 40% of the total protein is NifHDK^49^. For this, a simple protein solubility study is always the first experiment to perform before initiating more complex analyses^22,27^. From the 35 mitochondrial-targeted NifH variants expressed in this study, we obtained nine that were soluble in yeast mitochondria. The phyla from where these nine NifH variants originated were diverse, and so was their mechanisms of nutrition and relationship to oxygen. The only common factor we could observe was a bias towards coming from thermophilic organisms, as has been observed and discussed previously in works from our laboratory^22,24,27^.

To expand the analysis of these soluble NifH variants and to see if we could identify properties that would facilitate their functionality in future crops, we tested two aspects that are sought after for eukaryotic nitrogenase engineering; 1) simplification of the nitrogenase genetic machinery by minimizing the number of genes needed to transfer, and 2) identification of Nif components with better functionality in an environment containing oxygen. In this work, that meant (1) the identification of a NifH variant that did not depend on NifM for solubility and functionality, and (2) one NifH variant whose [Fe_4_S_4_] cluster was more resistant towards O_2_.

Although NifM is just one protein, each gene fewer to transfer will make the engineering of nitrogenase in plants less complex. When the *K. pneumoniae* NifH protein was expressed in *Escherichia coli* in the absence of NifM, the protein was much less stable and completely inactive^50^. This was in agreement with the low levels of NifH^*Kp*^ polypeptide and dinitrogenase reductase activity detected in *nifM*^−^ strains of the native host^51^. Work in yeast has shown that NifM co-expression was required for homodimer formation and polypeptide stability of *Rhizobium meliloti* NifH^52^, and in tobacco NifM was required to prevent NifH aggregation in the mitochondria^53^. While not many studies have investigated how NifM acts on NifH, sequence analysis suggests NifM to be a member of the rotamase family (PF00639) containing a PPIC-type PPIASE domain^54^. Prolyl isomerases (also known as peptidylprolyl isomerases or PPIases) are enzymes that accelerate protein folding by catalyzing the *cis–trans* isomerization of prolyl peptide bonds. This annotation is consistent with work identifying Pro^259^ in NifH from *A. vinelandii* as the prime target for NifM action^25^. In this work, seven of the final eight variants contained a proline residue at the site corresponding to Pro^259^ in *A. vinelandii*. The only exception was NifH from *Firmicutes bacterium*, but this protein was significantly shorter than the other NifH variants and therefore lacking this proline. However, the only two NifH variants that showed NifM-dependent solubility (*Sc*NifH^*Gs*^ and *Sc*NifH^*Ht*^) corresponded to those that originated from organisms containing genes with some similarity to *nifM*^*Av*^. Therefore, our work suggests that presence of a *nifM* homologue in the organism genome is a better indicator of NifM-dependency than presence of a proline at a site corresponding to Pro^259^ in *A. vinelandii*.

Equally important is the identification of more O_2_-resistant nitrogenase components, as O_2_-sensitivity is likely to be the major barrier to overcome to obtain a functional plant nitrogenase^5^. Active NifH could be expressed in the cytosol of anaerobically cultured yeast, while only the mitochondria could produce active protein under aerobic conditions^33^. This has been explained by the low O_2_ concentration in the mitochondria of actively respiring cells. Whether it is possible to obtain similarly O_2_-depleted conditions in plant mitochondria is not known. One scenario would be to limit nitrogenase expression to plant cells in hypoxic niches^55^. In any case, the identification of more O_2_-tolerable nitrogenase components would be a breakthrough for the engineering of nitrogenase in crops. In this regard, we were surprised to see increased resistance towards O_2_ by NifH from *G. sulfurreducens*. As all NifH proteins contain a [Fe_4_S_4_] cluster, we assumed that the variants tested in this study would also show similar O_2_-susceptibility. Whether the [Fe_4_S_4_] cluster in *Sc*NifH^*Gs*^ is less exposed, or whether it is stabilized by other means, is not known but these are interesting questions for future work. Importantly, this study shows that Nif proteins with better properties for expression in eukaryotic cells exist in Nature, and that their identification could pave the way for the engineering of N_2_ fixing crops.

## Materials and methods

### NifH library design and assembly

The majority of the *nifH* genes originated from a previously published gene set^22^. To this *nifH* library the genes for expression of NifH originating from *L. boryana*, *Frankia alni* and *D. ethenogenes* were added. All yeast codon-optimized DNA sequences and their corresponding protein products can be found in Supplementary Table S2. The *nifH* genes originating from the previously published gene set^22^ were amplified as *cox4-ts-nifH* gene fusions by PCR using primers #2584 (5´-AATTTTTGAAAATTCGAATTCCTCTTGACCATGCTTTCAC-3’) and #2585 (5´-GAAGAATTGTTAATTAAGAGCTCGGGGAAATTCGAGCTGG-3´). These primers include 15 bp overhangs complementary to the pESC-HIS yeast expression plasmid (#217451, Agilent Technologies) when digested with *Sac*I and *EcoR*I, and allowed for the insertion by an exonuclease and ligation-independent (ELIC) method^56^. The *nifH* genes originating from *L. boryana*, *F. alni* and *D. ethenogenes* were amplified using primers #2902 (5´-CACAATTTGAAAAAGGATCCATGTCTGACGAAAACATTAG-3´) and #2903 (5´-GGAAATTCGAGCTGGTCACCTTAAGCACCAGCCTTAGCCA-3´), #2904 (5´-CACAATTTGAAAAAGGATCCATGAGACAAATTGCTTTCTA-3´) and #2905 (5´-GGAAATTCGAGCTGGTCACCTTAAGCAACAGCAGCAGCCT-3´), #2906 (5´-CACAATTTGAAAAAGGATCCATGAGAAAGGTTGCTATTTA and #2907 (5´-GGAAATTCGAGCTGGTCACCTTAAGAAATAACACCAAATT-3´), respectively. The amplified *nifH* sequences were inserted by ELIC into pESC-HIS (*cox4-ts-nifH*^*B. japonicum*^) digested with *Nco*I and *BstE*II, replacing the *B. japonicum nifH* gene with *nifH* from *L. boryana*, *F. alni* or *D. ethenogenes*. The gene encoding for mitochondria-targeted SU9-NifM^*Av*^ was amplified from plasmid pN2XJ165^22^ using primers #2478 (5´- CTCTACAAATCTATCTCTCTCGAGATGGCCTCCACTCGTG-3´) and #2479 (5´- ATTATGGAGAAACTCGAGTTAACCATGTGCTAAGTTTTCC-3´) and inserted into pESC-TRP (#217453, Agilent Technologies) digested with *Xho*I by ELIC. The pESC-URA plasmid for expression of mitochondria-targeted Su9-NifU^*Av*^ and Su9-NifS^*Av*^ has been previously described^33^. All DNA digestions were performed using enzymes from New England Biolabs. PCR amplifications were carried out using Phusion Hot Start II High-Fidelity DNA Polymerase (ThermoFisher Scientific). ELIC products with their corresponding digested target vectors were transformed using a molar ratio 1:4 (vector:insert) into chemically competent *E. coli* DH5α and selected on solid LB (Lysogenic broth) media supplemented with appropriate antibiotics. Plasmid preparations were performed using Qiaprep Spin Miniprep kit (QIAGEN) and correct cloning was confirmed by Sanger sequencing (Macrogen). Plasmids were transformed into *S. cerevisiae* W303-1a (*MAT*a *leu2-3,112 trp1-1 can1-100 ura3-1 ade2-1 his3-11,15*) according to the lithium acetate method^57^, and selected and grown in synthetic drop-out medium with the appropriate auxotrophic selection^24^.

### Expression analysis and solubility screening of *Sc*NifH^Xx^ variants

Small-scale yeast protein extracts were prepared from yeast grown in galactose induction media as previously described^24^. The YeastBuster protein extraction reagent (Merck) was used to prepare total and soluble yeast protein extracts. First, galactose-induced yeast was pelleted for 10 min at 3000×*g*. YeastBuster mixture supplemented with 25 μg/ml DNAse I and 1 mM phenyl-methylsulfonyl fluoride (PMSF) was added to the yeast pellets at a ratio of 9 μl per OD × ml in Eppendorf tubes, and then incubated on a Eppendorf shaker for 20 min at room temperature to lyse the cells. This sample was then divided in two equal parts. For total extracts, the resulting YeastBuster lysate was added to 2 × Laemmli buffer at a 1:1 (v/v) ratio. For soluble extracts, the YeastBuster lysate was centrifuged in a benchtop centrifuge at maximum speed for 20 min at 4 °C before the supernatant was added to 2 × Laemmli buffer at a 1:1 (v/v) ratio. Both samples were prepared for SDS-PAGE by heating for 5 min at 95 °C.

Following SDS-PAGE, proteins were either stained using Coomassie brilliant Blue R-250 (Sigma) or transferred to nitrocellulose membranes (Protran Premium 0.45 µm, GE Healthcare) membranes for immunoblotting. Nitrocellulose membranes were stained with Ponceau S (Sigma) to ensure equal loading control and successful transfer. The membranes were blocked with 5% non-fat milk in TBS-T (20 mM Tris–HCl pH 7.5, 150 mM NaCl, 0.02% Tween-20) for 1 h at room temperature before incubation with primary antibodies overnight at 4 °C. Polyclonal antibodies detecting NifM^*Av*^ (used at 1:2,000 in 5% BSA), NifU^*Av*^ (used at 1:2000 in 5% BSA) and NifS^*Av*^ (used at 1:1,000 in 5% BSA), were raised against purified preparations of the corresponding *A. vinelandii* proteins (generated in house). Strep-tag II antibody (“Strep-MAB”, IBA Lifesciences, 1:2000 in 5% BSA) was used for detection of all *Sc*NifH^*Xx*^ variants. Secondary antibodies (Sigma) were diluted 1:20,000 in TBS-T supplemented with 2% non-fat milk and incubated for 2 h at room temperature. Membranes were developed using enhanced chemiluminescence and images were recorded digitally (iBright FL1000, ThermoFisher).

### *S. cerevisiae* growth and NifH variants purification

The growth of yeast cultures, galactose-induced Nif expression and STAC-purification of soluble *Sc*NifH^Xx^ variants followed the procedure previously described^27^. Cell pellets from 4 l fermenters stored in liquid N_2_ (typically 200–220 g) were resuspended in lysis buffer (100 mM Tris–HCl pH 8.8, 200 mM NaCl, 10% glycerol, 2 mM DTH, 1 mM PMSF, 1 μg/ml leupeptin, 5 μg/ml DNAse I) at a ratio of 1:2 (w/v) inside an anaerobic glovebox (Coy Laboratories). Total extracts (TE) were prepared by lysis of the cell suspensions under anaerobic atmosphere using an EmulsiFlex-C5 homogenizer (Avestin Inc.) operating at 20,000 psi. The TE was transferred to centrifuge tubes equipped with sealing closures (Beckman Coulter) and centrifuged at 50,000×*g* for 1 h at 4 °C (Avanti J-26 XP). The supernatant was filtered using filtering cups with a pore size of 0.2 μm (ThermoFisher), rendering cell-free extract (CFE) of soluble proteins that was loaded at 2.5 ml/min into a 5 ml Strep-Tactin XP column (IBA LifeSciences) attached to an ÄKTA FPLC (GE Healthcare) at O_2_-levels below 1 ppm in anaerobic chambers operating at 16 °C (MECAPLEX or MBraun). The column was washed overnight using about 120 ml wash buffer (100 mM Tris–HCl pH 8.0, 200 mM NaCl, 10% glycerol, 2 mM DTH). Strep-Tactin XP column-bound proteins were eluted with 15 ml washing buffer supplemented with 50 mM biotin (IBA LifeSciences). The elution fraction was concentrated using centrifugal filters with 30 kDa cutoff (Amicon, Millipore), loaded into PD-10 desalting columns (GE Healthcare) equilibrated with wash buffer to remove biotin and DTH, and then used to UV–Vis absorption spectrum analysis (see section below). The desalted eluate was supplemented with 2 mM DTH, further concentrated using centrifugal filters and finally snap-frozen as protein pellets in cryovials (Nalgene) and stored in liquid N_2_.

### Protein quantification, UV–Vis absorption spectrum and iron measurements

The concentrations of purified *Sc*NifH^*Xx*^ variants were measured using the BCA protein assay (Pierce) in combination with iodoacetamide to eliminate the interfering effect of DTH^58^. *Sc*NifH^*Xx*^ UV–Vis absorption spectra were recorded after removal of the DTH from the protein samples. The DTH-free protein samples were further diluted in wash buffer and transferred to Q6 spectroscopy cuvettes with sealing closures. Absorption (280 nm to 800 nm) was recorded using a UV-2600 spectrophotometer (Shimadzu). For recording of the air exposed *Sc*NifH^*Xx*^ samples, the sealing closure was removed, and the protein sample was carefully exposed to air using a pipette equipped with a gel loading tip. Iron content of as isolated *Sc*NifH^*Xx*^ preparations and NifH^*Av*^ (used as [Fe_4_S_4_] containing control protein) was determined by atomic absorption using a graphite furnace installed in a ContrAA 800 AAS Spectrometer (Analytik Jena). Protein samples were denatured using 30% HNO_3_ for 1 h at 80 °C, and then diluted in metal-free ultra-pure water to a final concentration of 1.5% HNO_3_. Twenty µl of diluted sample were used for iron measurement according to the following protocol: I) sample drying at 100 °C for 25 s, II) pyrolysis at 350 °C for 10 s, III) pyrolysis at 1100 °C for 20 s, IV) gas adaptation at 1100 °C for 5 s, V) atomization at 2000 °C for 10 s, and finally VI) cleaning of the furnace at 2500 °C for 5 s. Each sample was measured in triplicates. An absorbance wavelength of 248.327 nm was selected for specific iron measurement. For quantification, an iron standard curve from 0 to 20 parts per billion (ppb) was prepared from a 1000 parts per million (ppm) iron standard solution (Inorganic Ventures). The spectrometer protocol was set up and controlled using the ASpect CS software (version 2.2.2.0).

### Nitrogenase activity determination by S_2_V^red^ assay in cuvette

Nitrogenase activity of *Sc*NifH^*Ht*^ expressed in yeast mitochondria and isolated by STAC^22^ was determined following a recently described spectrophotometric method^21^. Activity assays were performed in cuvette (600 µl final reaction volume) in the presence of ATP regenerating mixture (6.7 mM MgCl_2_, 5 mM ATP, 30 mM phosphocreatine, 0.2 mg/ml creatine phosphokinase, 1.3 mg/ml bovine serum albumin (BSA) in 100 mM MOPS pH 7.0) and 0.5 mM 1,1′-bis(3-sulfonatopropyl)-4,4′-bipyridinium (S_2_V^red^). Nitrogenase activity was determined from the decrease in absorbance at 600 nm upon addition of 0.4 µM of NifDK^*Av*^ and increasing concentrations of NifH^*Av*^ or *Sc*NifH^*Ht*^. Absorbance was recorded using a USB 400-ISS-UV/VIS spectrophotometer (Ocean Optics) using a cuvette with a path length of 0.2 cm. Nitrogenase activity calculations were performed as previously described^21^.

### Nitrogenase activity determination by S_2_V^red^ assay in 96-well microtiter plates

Nitrogenase activity determined by the S_2_V assay were scaled down to 200 µl reaction volume to be performed in a 96-well microtiter plate. Except for during the optimization of the method, the assay was performed using a mixture of 2 µM NifH^*Av*^ and 0.05 µM NifDK^*Av*^ (corresponding to a NifH:NifDK ratio of 40:1). Other reaction conditions such as buffer composition, ATP regenerating mixture and S_2_V^red^ concentration were identical to those described in the section above. The plate was prepared under anaerobic conditions inside a glovebox (Coy Laboratories) and sealed using PCR plate sealing films. Absorbance reading was performed using an absorbance plate reader (SPECTROstar Nano, BMG LABTECH) operating at 30 °C. The absorbance was recorded for 1 h, with measurements taken every 30 s. Nitrogenase activity calculations were performed in Excel (Microsoft) using an molar extinction coefficient of 9925 M^−1^ cm^−1^ at 600 nm^21^, and a path length of 5 mm. The slope was calculated using the range in which the decrease in absorbance was linear, normally for at least 10 min. The final calculation can be expressed in simplified form as *k*_obs_ (s^−1^) =|m|/ ((ε∙l) ∙ [NifDK^*Av*^]), where |m| is the absolute value of the slope of the decrease in absorbance at 600 nm, ε is the molar extinction coefficient of S_2_V^red^ at 600 nm in M^−1^ cm^−1^ (9925), l is the path length in cm (0.5) and [NifDK^*Av*^] is the molar concentration of NifDK^*Av*^ in the assay (normally 0.05e^−6^).

### Nitrogenase activity determination by ARA

ARA were performed by combining isolated *Sc*NifH^*Xx*^ variants (2.2 µM) with pure NifDK^*Av*^ (0.055 µM) in an ATP regenerating mixture (1.23 mM ATP, 18 mM phosphocreatine disodium salt, 2.2 mM MgCl_2_, 3 mM DTH, 40 µg/mL creatine phosphokinase in 100 mM MOPS pH 7.0). The final reaction volume was 400 µl in 9 ml sealed vials. Vials were flushed with argon before the injection of 0.5 ml acetylene. After 15 min of incubation at 30 °C, reactions were stopped by the addition of 100 µl of 8 M NaOH. Ethylene produced was detected and quantified using a gas chromatograph (GC-2014, Shimadzu) fitted with a flame ionization detector. The separation column was a Porapak N 80/100 column (G3591-80072, Agilent technologies), using pure N_2_ as a column carrier gas (25 ml/min flow), and a mixture of H_2_/air for the flame.

### Oxygen sensitivity assays

*Sc*NifH^*Xx*^ samples and NifH^*Av*^ were passed through PD-10 desalting columns (GE Healthcare) equilibrated with 100 mM MOPS (pH 7.5) to remove DTH. Oxygen sensitivity was tested inside an anaerobic glovebox (Coy Laboratories) as previously described^31^ with slight modifications. First, 2.5 ml of 17.4 μM NifH in 100 mM MOPS (pH 7.5) was prepared in a 13 ml sealed glass vial. The atmosphere in the headspace was exchanged for argon. Diluted NifH sample was removed (t_0_) before pure O_2_ was injected (0.2 atm final) using a 250 μl gastight syringe (Hamilton). The vial was incubated at room temperature with shaking (800 rpm) on a thermomixer (Eppendorf) with an adaptor for 13 ml vials. Air exposed NifH samples were removed after 1, 2, 5 and 10 min. Fifty ul was transferred to open 9 ml vials (three technical replicates) containing 150 μl of 100 mM MOPS (pH 7.5) supplemented with 4 mM DTH. Finally, 200 ul ATP mixture supplemented with 5 µg of NifDK^*Av*^ was added before nitrogenase activity was measured following the protocol for ARA as described above.

## Supplementary Information

Supplementary Information 2.

Supplementary Information 3.

## Data Availability

The authors declare that the data supporting the findings of this study are available within the article, its supplementary information and data, and upon request.
