# Supplementary material for: A colorimetric method to measure in vitro nitrogenase functionality for engineering nitrogen fixation
Source: Sci Rep. 2022 Jun 20;12:10367. doi: 10.1038/s41598-022-14453-x (PMC9209457; doi:10.1038/s41598-022-14453-x)
Supplement: Supplementary file 2 — Supplementary Information 3. [file 41598_2022_14453_MOESM2_ESM.docx]

**Supplementary Information to**

**A colorimetric method to measure nitrogenase functionality for engineering nitrogen fixation**

Lucía Payá-Tormo, Diana Coroian, Silvia Martín-Muñoz, Artavazd Badalyan, Robert T. Green, Marcel Veldhuizen, Xi Jiang, Gema López-Torrejón, Janneke Balk, Lance C. Seefeldt, Stefan Burén & Luis M. Rubio

**Contents:**

**Figure S1**. Decrease in absorbance of S_2_V^red^ over time using distinct NifDK*^Av^* and NifH*^Av^* concentrations.

**Figure S2**. Change in *k*_obs_ over time using distinct NifDK*^Av^* and NifH*^Av^* concentrations.

**Figure S3**. Immunoblot analysis of *Sc*Nif*^Xx^* protein expression.

**Figure S4**. Purification of *Sc*NifH*^Xx^* variants.

**Figure S5**. UV-vis spectra of purified ScNifH^Xx^ variants.

**Figure S6**. Isolation of *Sc*NifH*^De^* expressed without NifM*^Av^*.

**Figure S7**. Alignment of NifH*^Av^* with the eight *Sc*NifH*^Xx^* variants.

**Figure S8**. NifM*^Av^* dependent *Sc*NifH*^Ht^* solubility and activity.

**Figure S9**. Uncropped immunoblots and membranes shown in Fig. S3.

**Figure S10**. Uncropped immunoblots and membranes shown in Fig. S3.

**Figure S11**. Uncropped Coomassie stained gel shown in Fig. 4c.

**Figure S12**. Uncropped immunoblots and membranes shown in Fig. 6a.

**Figure S13**. Uncropped gel shown in Fig. 6b.

**Figure S14**. Uncropped immunoblots and membranes shown in Fig. S8a-b.

**Table S1.** NifH expression plasmids and the yeast strains generated.

**Table S2.** Detailed information about the NifH library (Excel dataset)

**Supplementary references**.


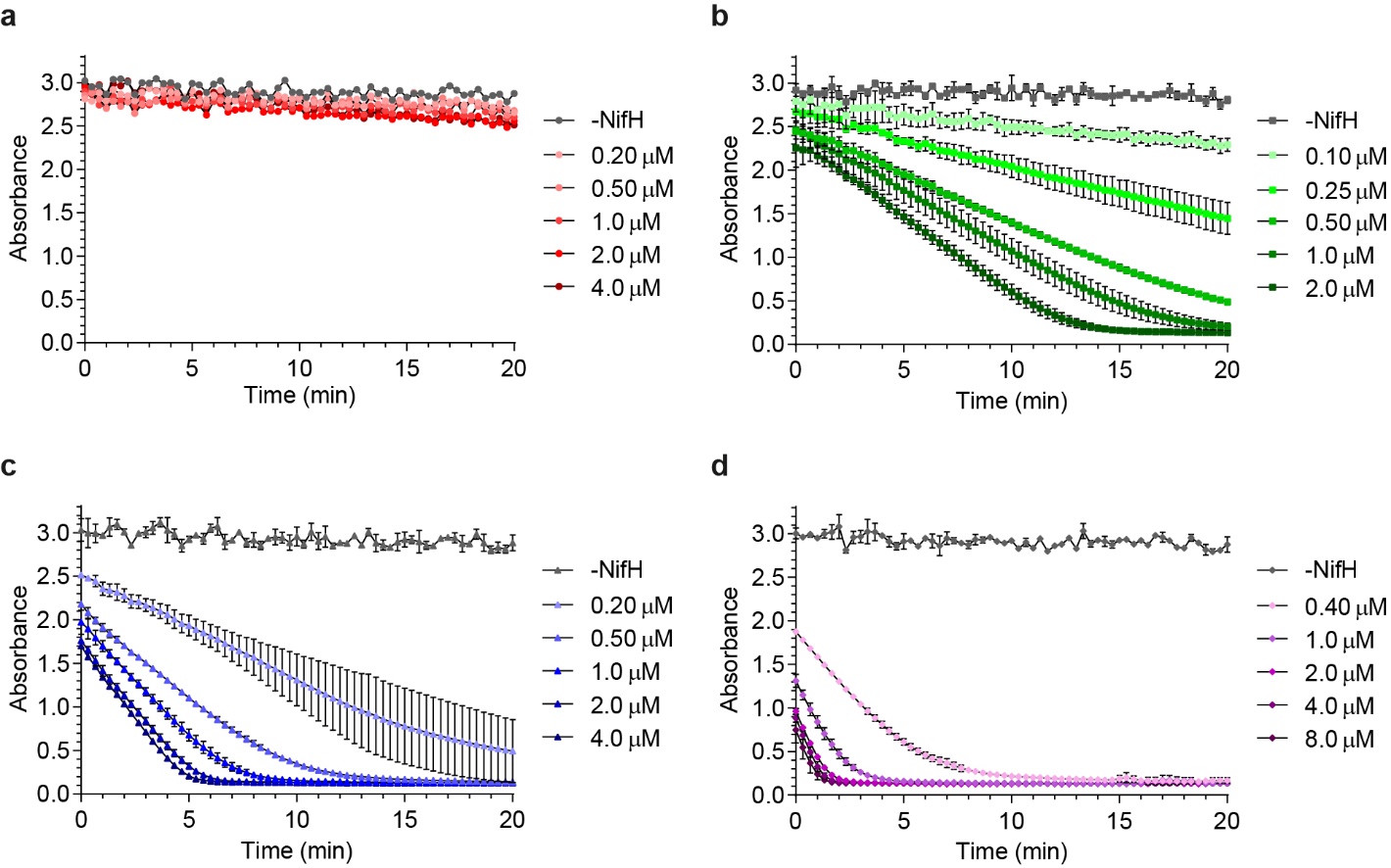


**Supplementary Figure S1.** Decrease in absorbance of S_2_V^red^ over time using distinct NifDK*^Av^* and NifH*^Av^* concentrations. **(a-d)** Oxidation of S_2_V^red^ as determined by reduction of absorbance at 600 nm using increasing NifH*^Av^* concentrations as specified on the right side of each panel in the absence of NifDK*^Av^* (a), or in the presence of 0.05 µM (b), 0.1 µM (c) or 0.2 µM (d) NifDK*^Av^*. Mean and SD is shown for *n* = 2 technical replicates.


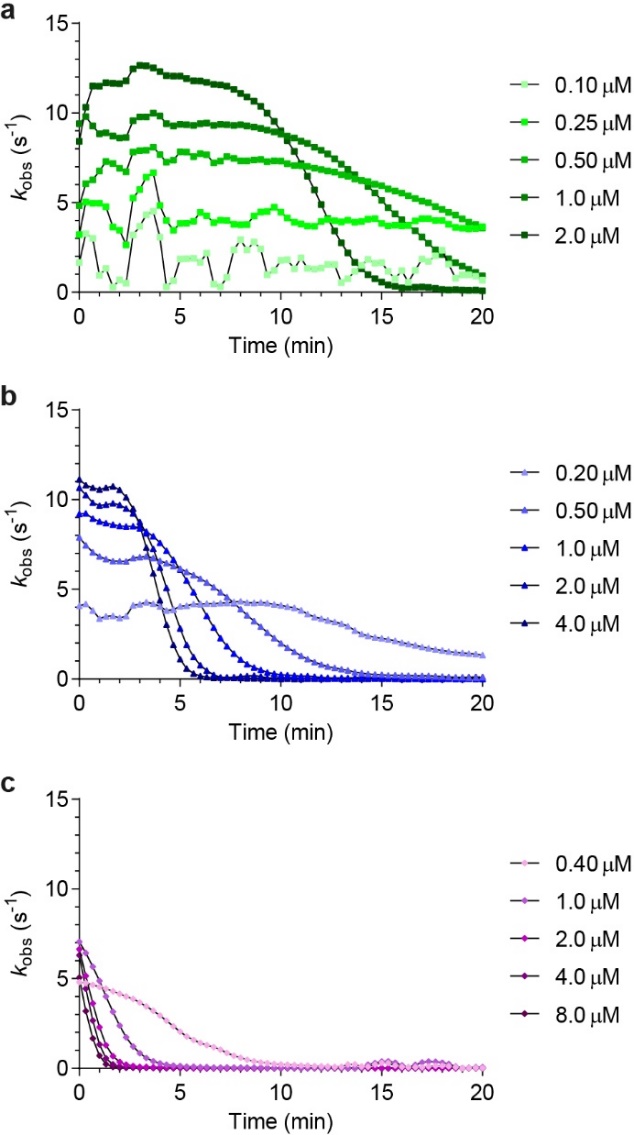


**Supplementary Figure S2.** Change in *k*_obs_ over time using distinct NifDK*^Av^* and NifH*^Av^* concentrations. (**a-c**) The decrease in absorbance of S_2_V^red^ shown in Supplementary Figure 1 was used to determine nitrogenase activity (*k*_obs_ (s^-1^)) in reactions containing 0.05 µM (a), 0.1 µM (b) or 0.2 µM (c) NifDK*^Av^*. The NifH*^Av^* concentrations are specified on the right side of each panel. Values show means for *n* = 2 technical replicates.


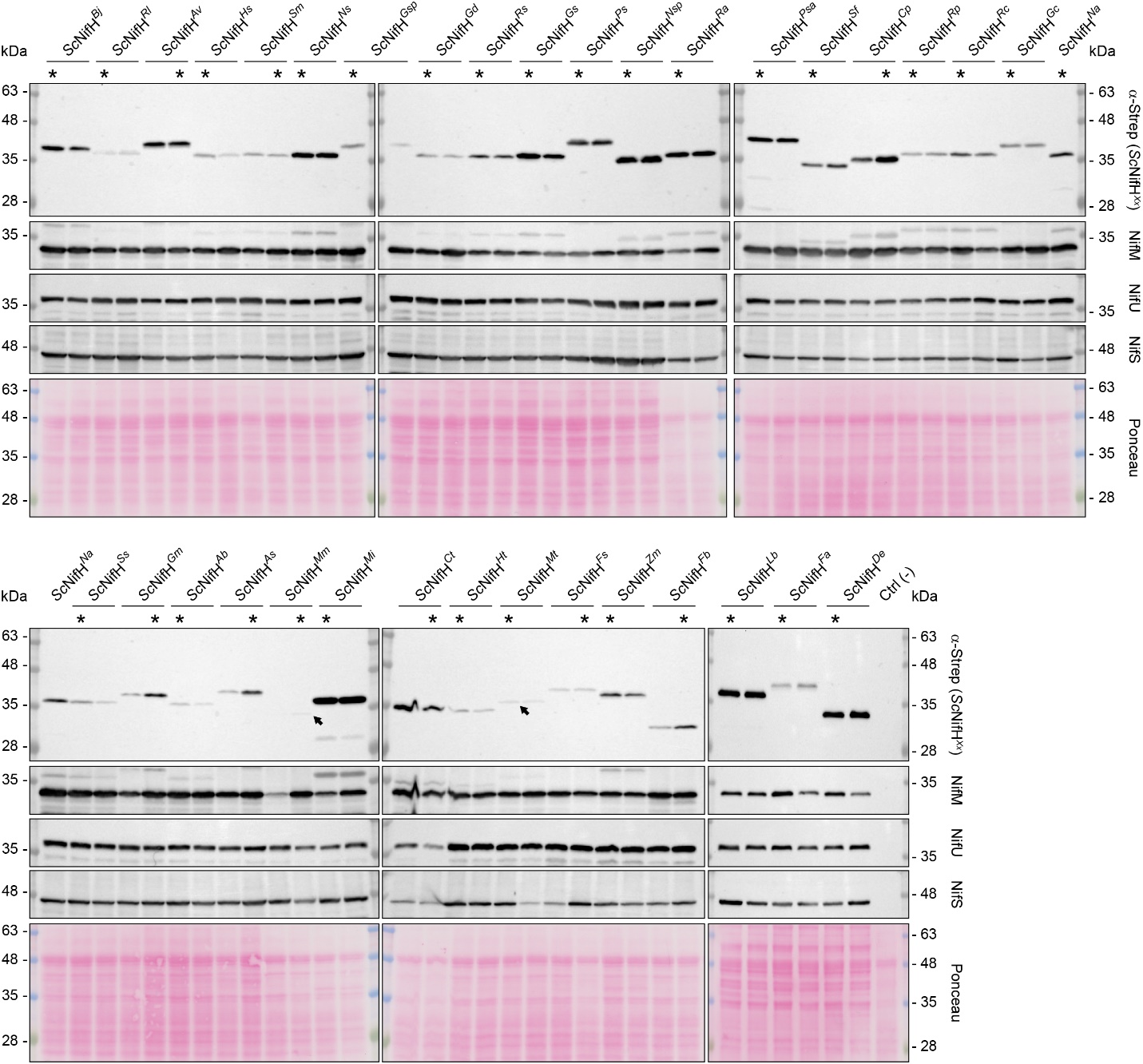


**Supplementary Figure S3.** Immunoblot analysis of *Sc*Nif*^Xx^* protein expression (see supplementary Table S2 for full species names). Total protein extracts were prepared from two clones of each NifH library yeast strain and analyzed for expression of TS-NifH (α-Strep), NifM, NifU and NifS. Clones marked with an asterisk were selected for solubility screening in Figure 3. Black arrows point at weakly expressed NifH variants. Total protein loading is shown by Ponceau staining of membranes. The uncropped immunoblots and gels are shown in Fig. S9 (upper part) and Fig. S10 (lower part).


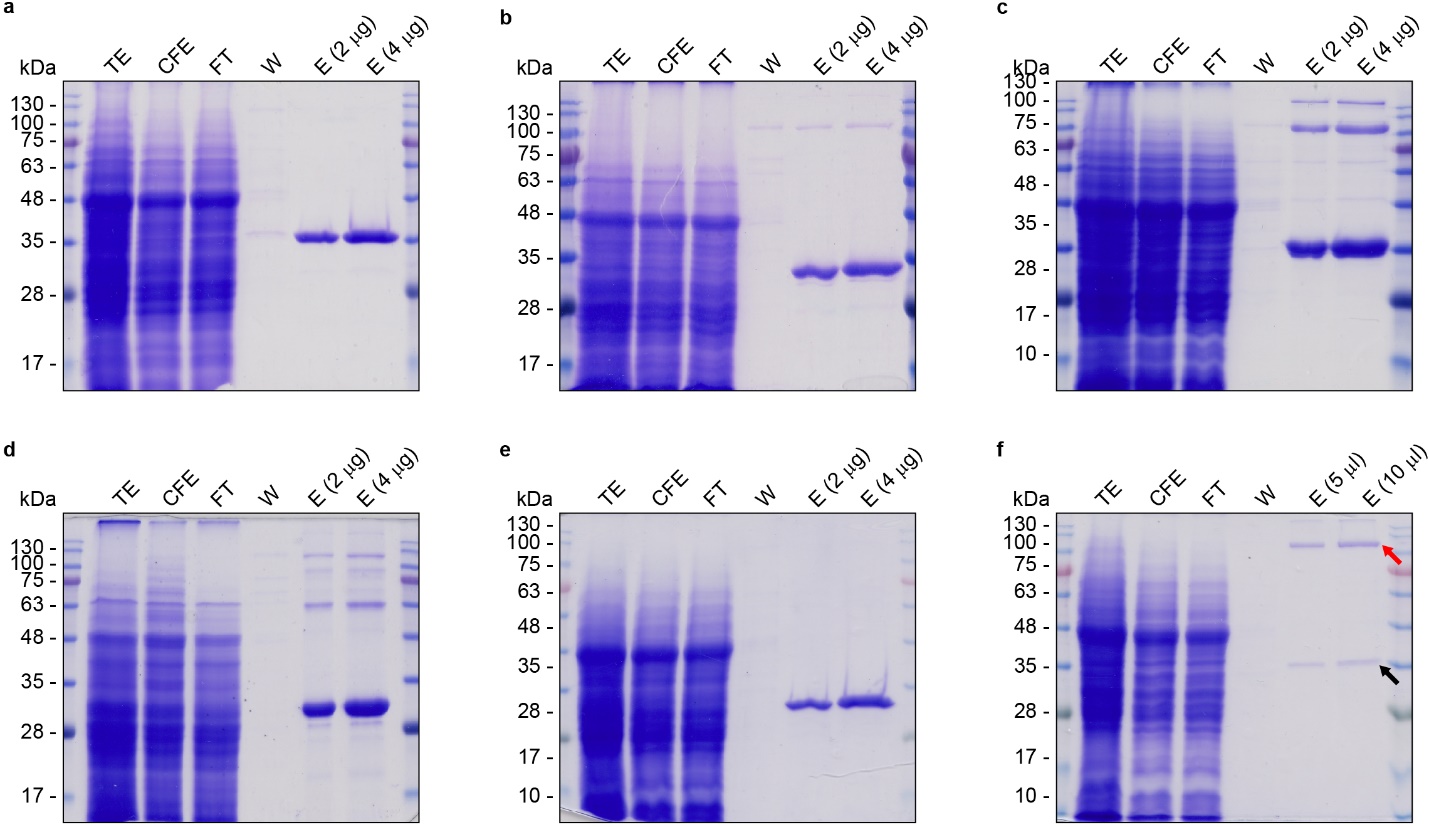


**Supplementary Figure S4.** Purification of *Sc*NifH*^Xx^* variants. **(a-f)** *Sc*NifH*^Rs^* (a), *Sc*NifH*^Ht^* (b), *Sc*NifH*^Gs^* (c), *Sc*NifH*^Fb^* (d), *Sc*NifH*^De^* (e) and *Sc*NifH*^Lb^* (f) were purified from mitochondria of aerobically cultured yeast using STAC. Larger volumes of the concentrated elution were loaded in (f) as low protein quantification precluded loading as in (a-e). TE, total extract after yeast cell breakage using high-pressure homogenizer; CFE, cell-free extract after centrifugation and filtering of the TE; FT, flow-through after passing the CFE through the STAC column; W, wash fraction; E, final concentrated and desalted elution fraction. The yield of *Sc*NifH*^Lb^* (f) was very low (black arrow), and most of the polypeptides in the elution fraction migrated at the size of pyruvate carboxylase (N1P377) that is sometimes present as contaminant in STAC purifications (red arrow)^1^. The purification of *Sc*NifH*^Ra^* is shown in Fig. 4a, and the purifications of *Sc*NifH*^Mm^* and *Sc*NifH*^Mi^* have previously been reported^1^.


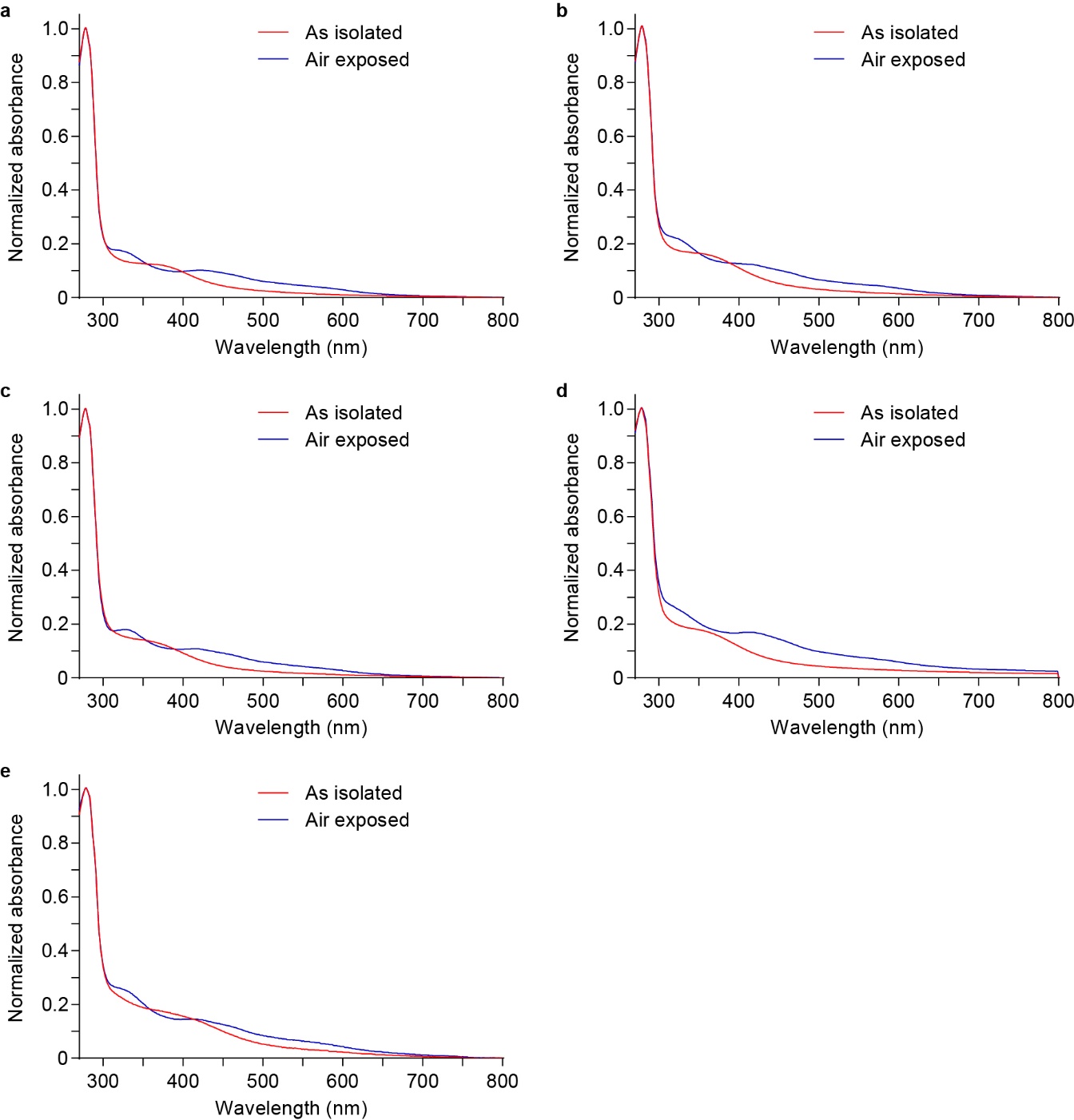


**Supplementary Figure S5.** UV-vis spectra of purified *Sc*NifH*^Xx^* variants shown in supplementary Fig. S4. **(a-e)** UV-vis absorption spectra were recorded in as-isolated and air-exposed *Sc*NifH*^Rs^* (a), *Sc*NifH*^Ht^* (b), *Sc*NifH*^Gs^* (c), *Sc*NifH*^Fb^* (d) and *Sc*NifH*^De^* (e) purified from yeast using STAC.


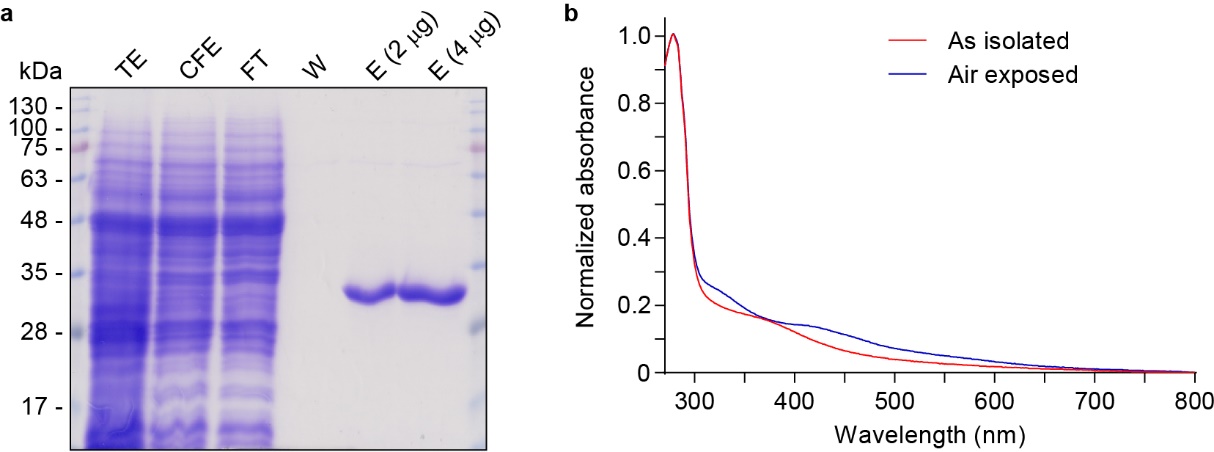


**Supplementary Figure S6.** Isolation of *Sc*NifH*^De^* expressed without NifM*^Av^*. **(a)** *Sc*NifH*^De^* was purified from mitochondria of aerobically cultured yeast using STAC. **(b)** UV-vis absorption spectra were recorded of as-isolated and air-exposed *Sc*NifH*^De^*.


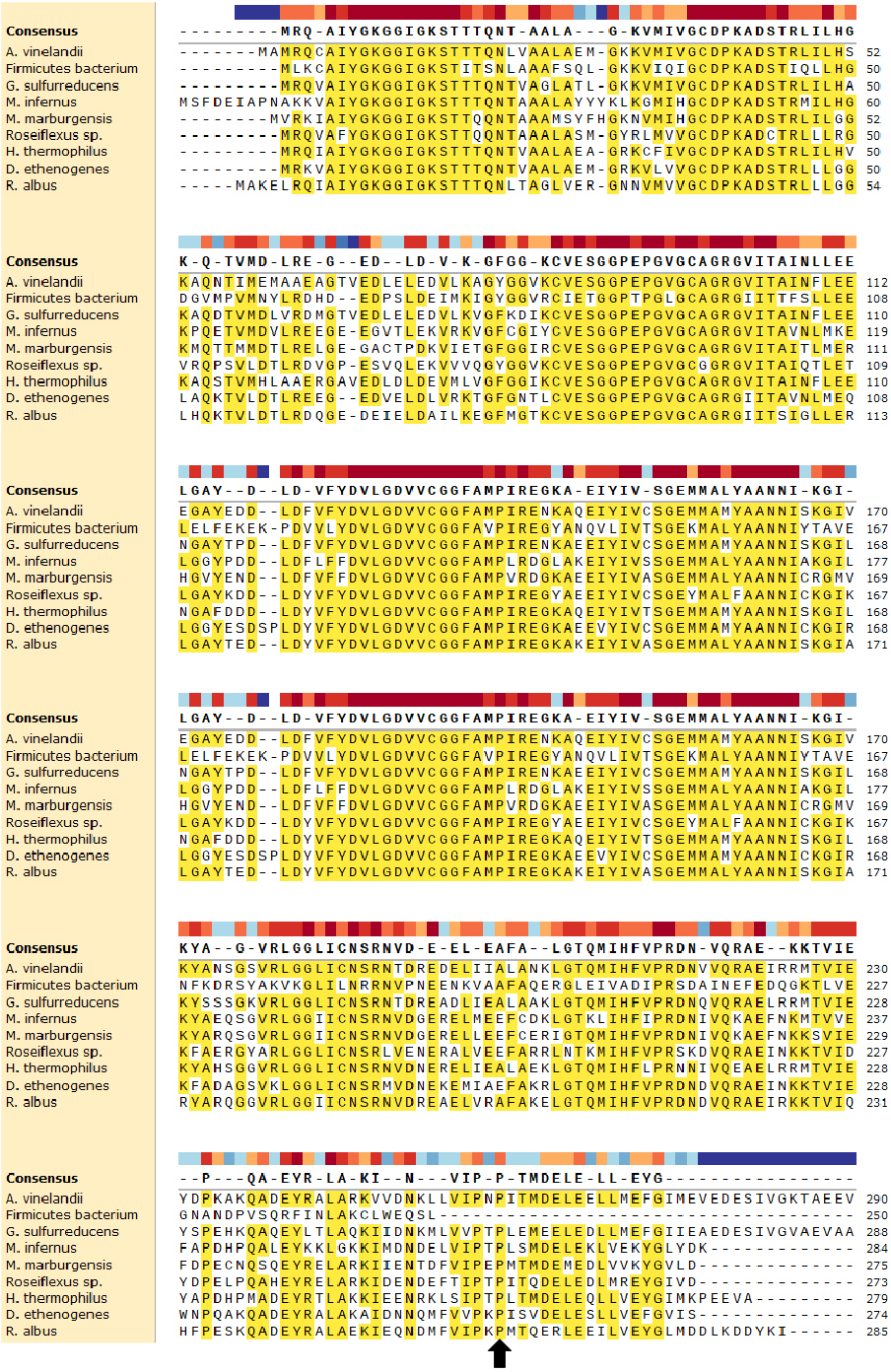


**Supplementary Figure S7.** Alignment of NifH*^Av^* with the eight *Sc*NifH*^Xx^* variants analyzed in Fig. 6. NifH protein sequence alignments. Multiple sequence alignment was performed in SnapGene (version 4.2.11) using the MUltiple Sequence Comparison by Log- Expectation (MUSCLE) method. The black arrow indicates the Pro^259^ in NifH*^Av^* proposed to be the target of NifM action.


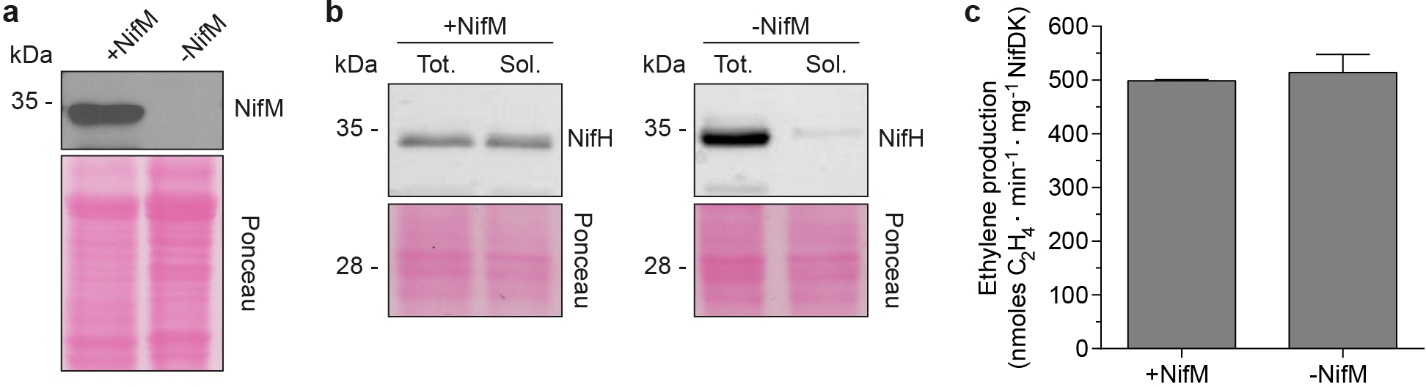


**Figure S8**. NifM*^Av^* dependent *Sc*NifH*^Ht^* solubility and activity. **(a)** Immunoblot analysis of yeast expressing (+NifM) or not expressing (-NifM) *Sc*NifM*^Av^*. Ponceau S staining was used as a loading control. The uncropped immunoblot and membrane are shown in Fig. S14. **(b)** Accumulation of *Sc*NifH*^Ht^* in total protein extracts (Tot.) and soluble protein extracts (Sol.) in yeast either co-expressing (+NifM) or not expressing (-NifM) *Sc*NifM*^Av^*. Ponceau S staining was used as a loading control. The uncropped immunoblots and membranes are shown in Fig. S14. **(c)** ARA using *Sc*NifH*^Ht^* isolated from yeast either co-expressing (+NifM) or not expressing (–NifM) *Sc*NifM*^Av^* using a 40:1 ratio of *Sc*NifH*^Ht^*:NifDK*^Av^*. The positive control ARA (40:1 ratio of NifH*^Av^*:NifDK*^Av^*) yielded 1961 ± 88 units. Mean and SD is shown. *n* = 2 technical replicates.


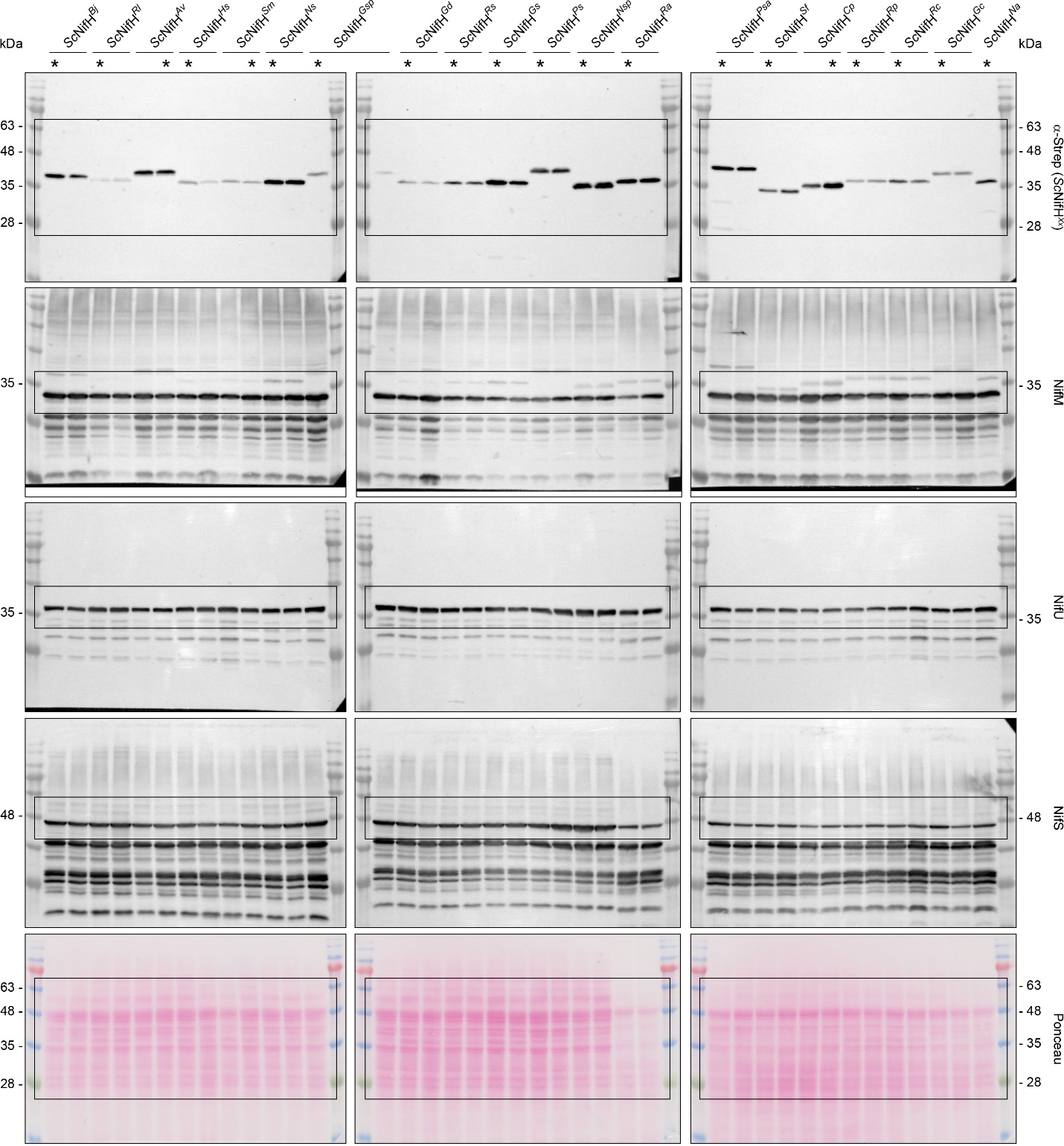


**Figure S9**. Uncropped immunoblots and membranes shown in Fig. S3. The upper part of Fig. S3 are shown. The membranes probed with Strep antibodies were reprobed with NifM antibody, and the membranes probed with NifU antibodies were reprobed with NifS antibody.


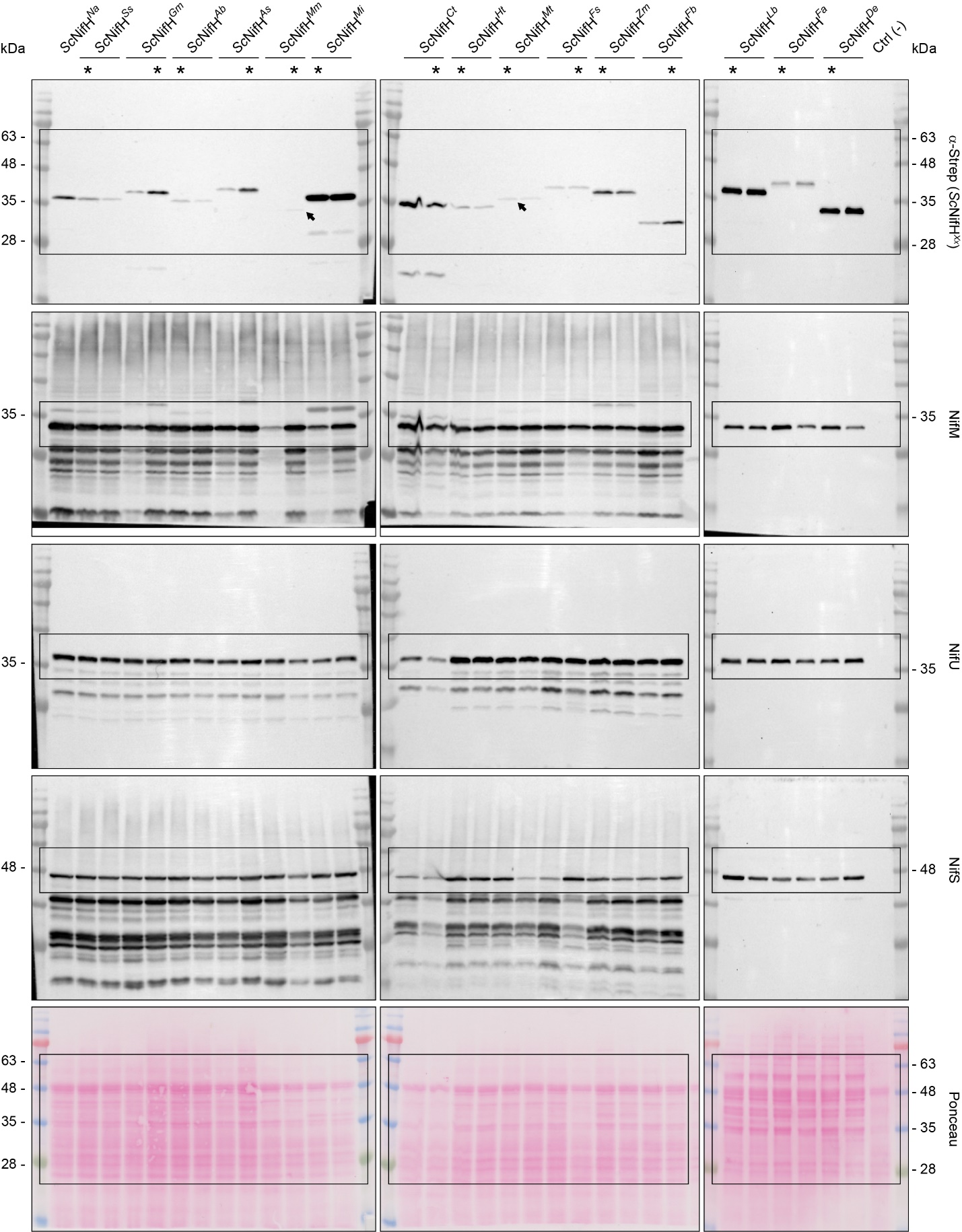


**Figure S10**. Uncropped immunoblots and membranes shown in Fig. S3. The lower part of Fig. S3 are shown. The membranes probed with Strep antibodies were reprobed with NifM antibody, and the membranes probed with NifU antibodies were reprobed with NifS antibody (except for the right column of immunoblots corresponding to protein extracts prepared from *Sc*NifH*^Lb^*, *Sc*NifH*^Fa^*, *Sc*NifH*^De^* and the negative control).


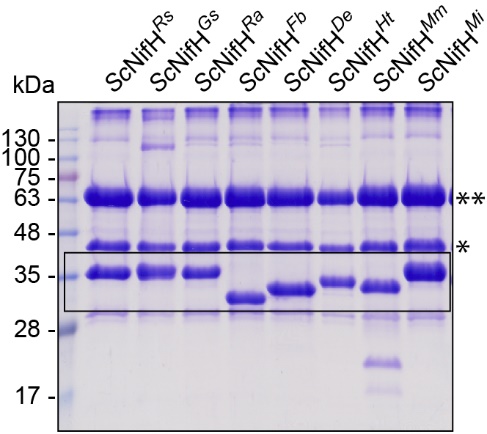


**Figure S11**. Uncropped Coomassie stained gel shown in Fig. 4c. The samples originate from the S_2_V^red^ nitrogenase assay shown in Fig. 4e. Coomassie stained polypeptides corresponding to creatine phosphokinase (*, ca. 43 kDa) and bovine serum albumin (**, ca. 66 kDa) present in the ATP regenerating mixture are indicated.


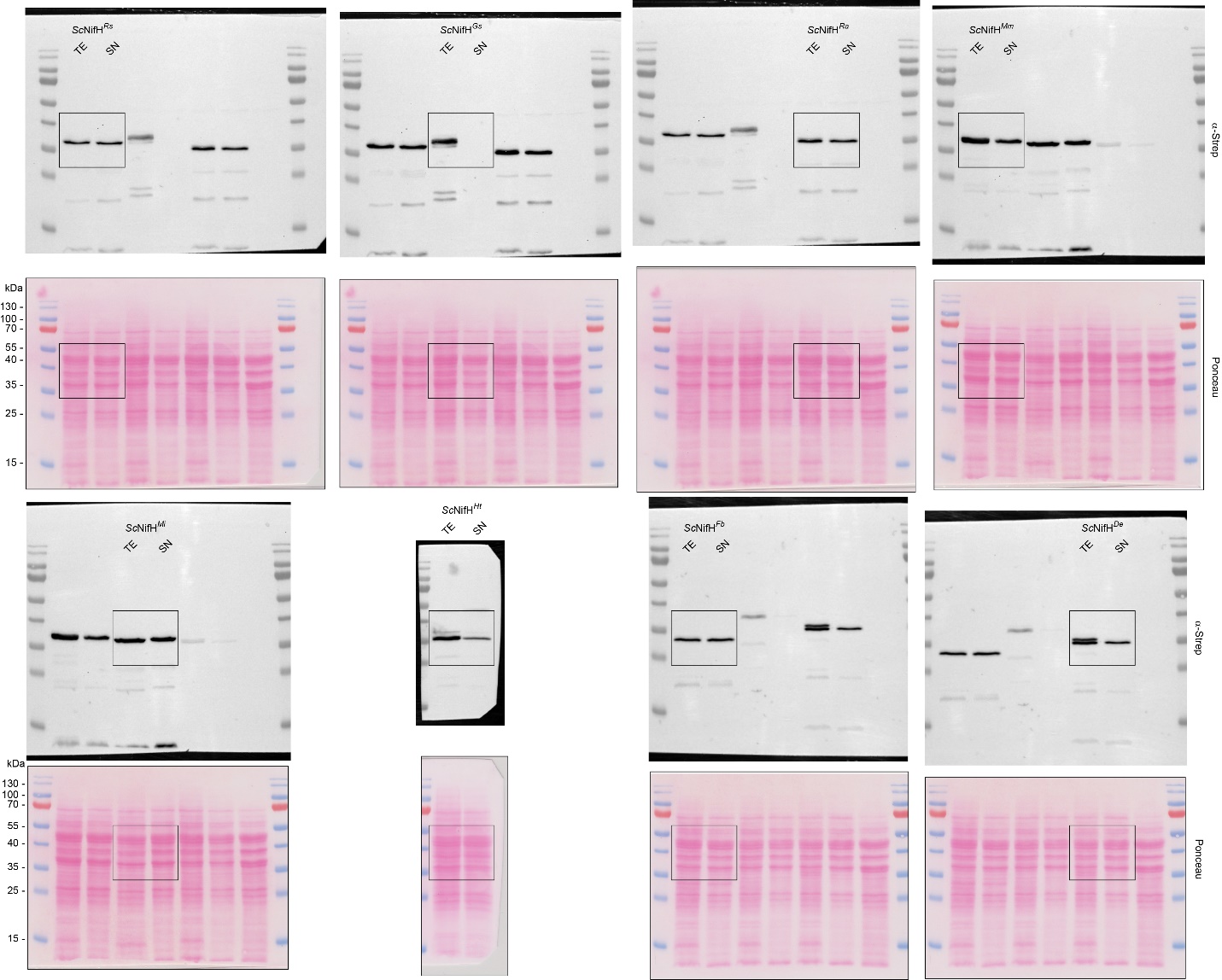


**Figure S12**. Uncropped immunoblots and membranes shown in Fig. 6a. The membrane for *Sc*NiH*^Ht^* was cut prior to hybridization with the Strep antibody.

**
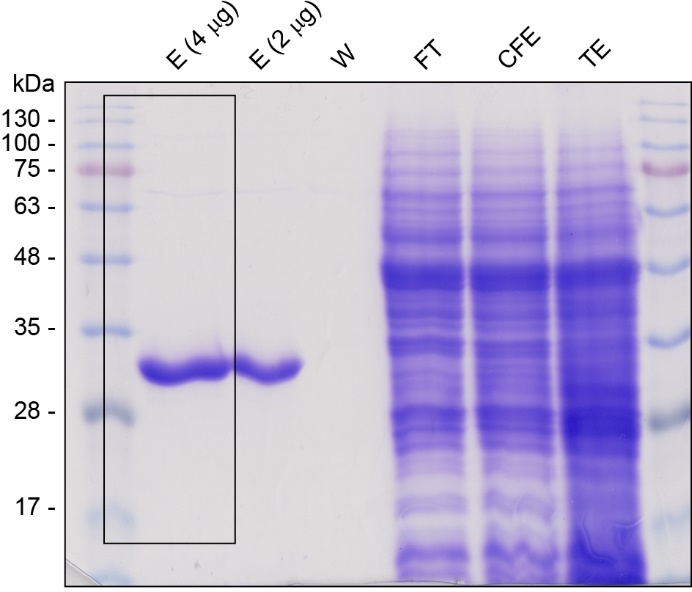
**

**Figure S13**. Uncropped gel shown in Fig. 6b. TE, total extract after yeast cell breakage using high-pressure homogenizer; CFE, cell-free extract after centrifugation and filtering of the TE; FT, flow-through after passing the CFE through the STAC column; W, wash fraction; E, final concentrated and desalted elution fraction.


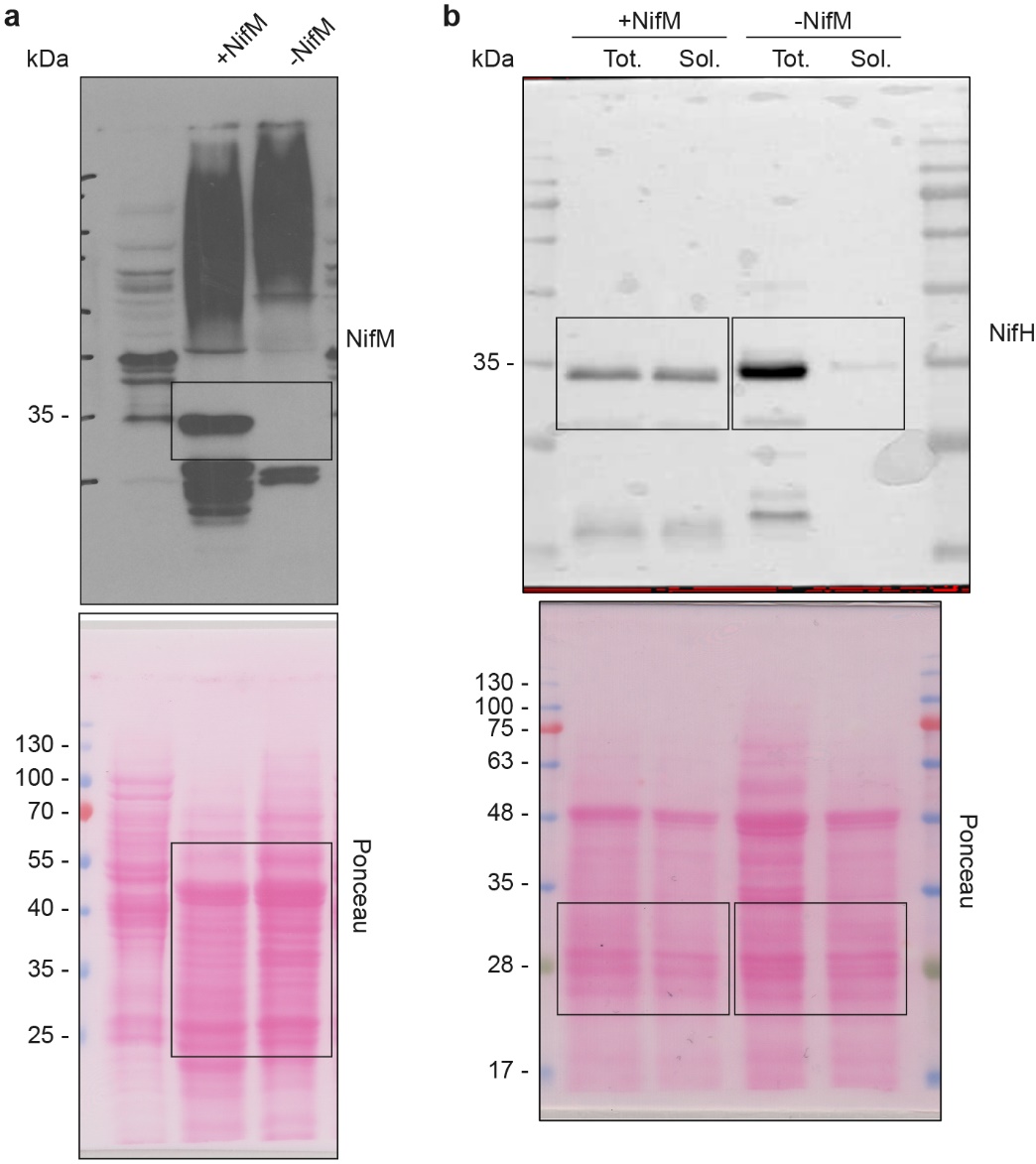


**Figure S14**. Uncropped immunoblots and membranes shown in Figure S8a-b.

**Supplementary Table S1.** NifH expression plasmids and the yeast strains generated.

| **NifH plasmid** | **Yeast strain** | **NifH variant** |
| --- | --- | --- |
| pN2LP83 | SB283Y | *Bradyrhizobium japonicum (strain USDA 6)* |
| pN2LP84 | SB284Y | *Rhizobium leguminosarum bv. trifolii (strain WSM1689)* |
| pN2LP85 | SB285Y | *Azotobacter vinelandii (strain DJ / ATCC BAA-1303)* |
| pN2LP86 | SB286Y | *Herbaspirillum seropedicae (strain SmR1)* |
| pN2LP87 | SB287Y | *Sinorhizobium meliloti (strain SM11)* |
| pN2LP88 | SB288Y | *Nostoc sp. (strain PCC 7120 / SAG 25.82 / UTEX 2576)* |
| pN2LP89 | SB289Y | *Gloeothece sp. KO68DGA* |
| pN2LP90 | SB290Y | *Gluconacetobacter diazotrophicus (strain ATCC 49037 / DSM 5601 / PAl5)* |
| pN2LP91 | SB291Y  SB321Y (-NifM) | *Roseiflexus sp. (strain RS-1)* |
| pN2LP92 | SB292Y  SB322Y (-NifM) | *Geobacter sulfurreducens (strain ATCC 51573 / DSM 12127 / PCA)* |
| pN2LP93 | SB293Y | *Pseudomonas stutzeri (strain DSM 4166 / CMT.9.A)* |
| pN2LP94 | SB294Y | *Nostoc sp. (strain PCC 6720) (Anabaenopsis circularis)* |
| pN2LP95 | SB295Y  SB323Y (-NifM) | *Ruminococcus albus (strain SY3)* |
| pN2LP96 | SB296Y | *Paenibacillus sabinae (strain T27)* |
| pN2LP97 | SB297Y | *Syntrophobacter fumaroxidans (strain DSM 10017 / MPOB)* |
| pN2LP98 | SB298Y | *Clostridium pasteurianum (strain BC1)* |
| pN2LP99 | SB299Y | *Rhodopseudomonas palustris* |
| pN2LP100 | SB300Y | *Rhodobacter capsulatus (strain ATCC BAA-309 / NBRC 16581 / SB1003)* |
| pN2LP101 | SB301Y | *Gloeothece citriformis (strain PCC 7424)/ Cyanothece sp. PCC 7424* |
| pN2LP102 | SB302Y | *Nostoc azollae (strain 0708) (Anabaena azollae (strain 0708))* |
| pN2LP103 | SB303Y | *Synechococcus sp. (strain JA-2-3B'a(2-13)) (Cyanobacteria bacterium Yellowstone B-Prime)* |
| pN2LP104 | SB304Y | *Geobacter metallireducens (strain GS-15 / ATCC 53774 / DSM 7210)* |
| pN2LP105 | SB305Y | *Azospirillum brasilense* |
| pN2LP106 | SB306Y | *Azoarcus sp. (strain BH72)* |
| pN2LP107 | SB307Y  SB324Y (-NifM) | *Methanothermobacter marburgensis (strain ATCC BAA-927 / DSM 2133 / JCM 14651 / NBRC 100331 / OCM 82 / Marburg) (Methanobacterium thermoautotrophicum)* |
| pN2LP108 | SB308Y  SB325Y (-NifM) | *Methanocaldococcus infernus (strain DSM 11812 / JCM 15783 / ME)* |
| pN2LP109 | SB309Y | *Chlorobium tepidum (strain ATCC 49652 / DSM 12025 / NBRC 103806 / TLS)* |
| pN2XJ203 | SB310Y  SB326Y (-NifM) | *Hydrogenobacter thermophilus (strain DSM 6534 / IAM 12695 / TK-6)* |
| pN2LP110 | SB311Y | *Methanothermobacter thermautotrophicus (strain ATCC 29096 / DSM 1053 / JCM 10044 / NBRC 100330 / Delta H) (Methanobacterium thermoautotrophicum)* |
| pN2LP111 | SB312Y | *Frankia sp. (strain FaC1)* |
| pN2LP112 | SB313Y | *Zymomonas mobilis subsp. mobilis (strain ATCC 31821 / ZM4 / CP4)* |
| pN2LP113 | SB314Y  SB327Y (-NifM) | *Firmicutes bacterium CAG:536* |
| pN2SB146 | SB316Y  SB328Y (-NifM) | *Leptolyngbya boryana (strain Dg5)* |
| pN2SB147 | SB317Y | *Frankia alni (strain ACN14A)* |
| pN2SB148 | SB318Y  SB329Y (-NifM) | *Dehalococcoides ethenogenes (strain 195) (Dehalococcoides mccartyi 195)* |

**Supplementary Table S2.** Detailed information about the NifH library (Excel dataset).

**Supplementary references**

1 Jiang, X. *et al.* Exploiting genetic diversity and gene synthesis to identify superior nitrogenase NifH protein variants to engineer N_2_-fixation in plants. *Commun Biol* **4**, 4, doi:10.1038/s42003-020-01536-6 (2021).
